# Topical emollient therapy with sunflower seed oil alters the skin microbiota of young children with severe acute malnutrition in Bangladesh: A randomised, controlled study

**DOI:** 10.7189/jogh.11.04047

**Published:** 2021-07-17

**Authors:** Natalie Fischer, Gary L Darmstadt, KM Shahunja, Jonathan M Crowther, Lindsay Kendall, Rachel A Gibson, Tahmeed Ahmed, David A Relman

**Affiliations:** 1 Division of Infectious Diseases & Geographic Medicine, Department of Medicine, Stanford University School of Medicine, Stanford, California, USA; 2 Prematurity Research Center, Department of Pediatrics, Stanford University School of Medicine, Stanford, California, USA; 3 Nutrition and Clinical Services Division, International Centre for Diarroheal Disease Research Bangladesh (icddr,b), Dhaka, Bangladesh; 4 JMC Scientific Consulting Ltd, Egham, Surrey, UK; 5 GlaxoSmithKline R&D, Global Health Catalyst, Stevenage, UK; 6 Department of Microbiology & Immunology, Stanford University School of Medicine, Stanford, California, USA; 7 Infectious Diseases Section, Veterans Affairs Palo Alto Health Care System 154T, Palo Alto, California, USA

## Abstract

**Background:**

Topical emollient therapy with sunflower seed oil (SSO) reduces risk of sepsis and mortality in very preterm infants in low- or middle-income countries (LMICs). Proposed mechanisms include modulation of skin and possibly gut barrier function. The skin and gut microbiota play important roles in regulating barrier function, but the effects of emollient therapy on these microbiotas are poorly understood.

**Methods:**

We characterised microbiota structure and diversity with 16S rRNA gene amplicon sequence data and ecological statistics in 20 children with severe acute malnutrition (SAM) aged 2-24 months, at four skin sites and in stool, during a randomised, controlled trial of emollient therapy with SSO in Bangladesh. Microbes associated with therapy were identified with tree-based sparse discriminant analysis.

**Results:**

The skin microbiota of Bangladeshi children with SAM was highly diverse and displayed significant variation in structure as a function of physical distance between sites. Microbiota structure differed between the study groups (*P* = 0.005), was more diverse in emollient-treated subjects–including on the forehead which did not receive direct treatment–and changed with each day (*P* = 0.005) at all skin sites. Overall, *Prevotellaceae* were the most differentially affected by emollient treatment; several genera within this family became more abundant in the emollient group than in the controls across several skin sites. Gut microbiota structure was associated with sample day (*P* = 0.045) and subject age (*P* = 0.045), but was not significantly affected by emollient treatment (*P* = 0.060).

**Conclusions:**

Emollient therapy altered the skin microbiota in a consistent and temporally coherent manner. We speculate that therapy with SSO enhances skin barrier function in part through alterations in the microbiota, and through systemic mechanisms. Strategies to strengthen skin and gut barrier function in populations at risk, such as children in LMICs like Bangladesh, might include deliberate manipulation of their skin microbiota.

**Trial registration:**

ClinicalTrials.gov: NCT02616289.

Over the last century, progress in sanitation, development of vaccines and the discovery of antibiotics greatly improved global health and enabled a drastic decrease in mortality due to communicable diseases [1]. The 58% decrease in under-five mortality from 93 deaths per 1000 live births in 1990 to 39 per 1000 in 2017 is a major measure of this progress [2]. In Bangladesh, the under-five mortality rate is still above the global average, with 45 deaths per 1000 live births [3]. Notably, nearly 45% of these child deaths are attributable to various forms of undernutrition, including severe acute malnutrition (SAM) which is defined by the World Health Organization as a weight-for-height/length Z score more than three standard-deviations below the median [4]. Moreover, acute infectious diarrhea and other infectious diseases contribute significantly to morbidity and mortality in SAM patients and are tightly intertwined with nutritional status and weight loss [5].

Topical emollient therapy was shown to lead to a significant increase in weight gain and reduction in mortality caused by bloodstream infections in very preterm infants [6,7]. A study in very preterm infants in Bangladesh found a 41% reduction in nosocomial infections after they received topical therapy with sunflower seed oil (SSO) as compared to untreated controls [8]. Furthermore, the barrier function of the skin was significantly improved by SSO therapy, as measured by improvement in skin condition [9] and by reduction in trans-epidermal water loss (TEWL) in a mouse model of human infant skin [10].

The skin of infants undergoes terminal differentiation and significant change in structure during the first year of life, enabling the colonisation of selected microbial species [11]. Spatial biogeography of the skin microbiota has been described in healthy Western infants and adults and correlated with site-specific biochemical and biophysical properties – in particular, ‘moist’, ‘dry’ or ‘sebaceous’ sites [12-14]. In addition to the physical and chemical barrier of the skin, the resident microbiota prevents colonisation by pathogens and helps educate the immune system [15]. Studies on the effects of emollients on skin microbiota structure are few, and most such studies focus on synthetic emollients used in Western medicine for the treatment of atopic dermatitis, as well as the role of microbes in the genesis of atopic lesions [16-18].

Besides the direct impact of oil on the skin, there is also evidence for systemic effects of emollients. Several studies in infants and in children with SAM have reported absorption through the skin and systemic distribution of essential fatty acids (EFAs) contained in emollients [19-21]. Studies in rodents indicate that EFAs modulate inflammation, improve gut barrier function and reduce gut-derived sepsis [22-24]. Malnutrition has been associated with inflammation of the gut epithelium, which can have detrimental effects on gut architecture, causing impaired nutrient absorption and increased permeability for microbes and microbial components, one of the underlying causes of environmental enteric dysfunction [25,26]. Circulation of microbial cell wall components in the bloodstream causes systemic inflammation, which increases energy expenditure, thus exacerbating malnutrition [27].

A clinical trial was initiated in order to learn whether emollient therapy might also benefit children with SAM [28], in addition to premature infants. Within this trial, we conducted a sub-study of the microbiome, which is presented here, based on the hypothesis that the previously observed improvements in skin barrier function and reductions in infection [28] as well as absorption of fatty acids [21] were linked to changes in skin and gut microbiota structure. Thus, we sought to assess the impact of daily topical massage with sunflower seed oil on the diversity and composition of the skin and gut microbiota, and secondarily address the relative lack of information about the structure of the skin microbiota in non-Western children.

## METHODS

### Study population

Twenty children aged 2-24 months who presented to the icddr,b Dhaka Hospital, Bangladesh, with SAM (weight-for-length Z score (WLZ)<-3 with or without the presence of nutritional edema) were enrolled in this microbiota sub-study of a clinical trial on the impact of topical emollient therapy for SAM (ClinicalTrials.gov identifier: NCT02616289) [28]; these twenty children were the last twenty participants to be enrolled in the trial. Children were randomised in a 1:1 ratio to: a) SAM routine standard-of-care alone, the control group (ten participants), or b) SAM routine standard-of-care + topical application of high linoleic acid (>60%) SSO by whole-body massage, three times daily with 3g of oil per kg of body weight per application, for ten days, the emollient group (ten participants), as described previously (see Appendix S1 in the **Online Supplementary Document**) [28]. No oil was applied over the face and head to reduce the possibility of oil aspiration or contact with the eyes. All patients received a comparable antibiotic regimen (see Figure S1, Table S1 in the **Online Supplementary Document**). Upon admission, children were started on ampicillin (100 mg/kg per day i.v. in 6-hourly doses for two days) and gentamicin (5 mg/kg per day i.v. or i.m. in 12-hourly doses for seven days). Ampicillin was followed by oral amoxicillin (for five days). Additional antibiotics such as azithromycin, cefixime, ceftriaxone, pivmecillinam or ciprofloxacin were given in a few cases based on the patient’s clinical condition and the physician’s judgment.

### Ethical considerations

Caregivers of all patients provided written informed consent prior to participation under approved human subjects protocols by the Institutional Review Boards at icddr,b (protocol #PR-15101) and at Stanford University, Stanford, California, USA (protocol #34646).

Trial registration: ClinicalTrials.gov: NCT02616289.

### Sample collection

Skin swabs and stool samples were collected at baseline from all twenty study participants upon admission to the hospital and enrollment in the study, prior to administration of antibiotics and before initiation of emollient therapy in the emollient group. The volar forearm and shin were chosen as representative ‘dry’ areas, the elbow crease as a ‘moist’ area, and the forehead as a ‘sebaceous’ area of the skin. During the following ten-day treatment period, skin swab samples were collected daily in the morning, to allow for the longest period after the last application of oil the previous evening and before application of oil for the new day. Swabs from the control group were collected on the same schedule. Stool samples in both treatment groups were collected daily. Two baseline stool samples were collected from 17 participants. One participant in the control group left after day five against medical advice and therefore did not complete the full sample collection scheme. In total, 232 stool samples and 859 skin swab samples were collected (Figure S2 in the **Online Supplementary Document**).

### 16S rRNA gene amplicon sequencing

All samples were randomised for DNA extraction as well as for PCR. DNA extraction, 16S rRNA gene V4 region amplification, and amplicon pool preparation and sequencing are described in Supplemental Methods. A total of 391 682 822 high quality 250-nucleotide reads were generated from the 232 stool samples and 859 skin swabs ( ~ 324 570 reads/sample, sd 33 160 reads) with the HiSeq 2500 (Illumina, San Diego, California, USA) sequencing platform.

### Data processing

Sequence demultiplexing, quality filtering, inference of amplicon sequence variants (ASVs), taxonomy assignment, and the building of a phylogenetic tree are described in Supplementary Methods in the **Online Supplementary Document**. The ASV table, patient sample data, taxonomy assignments, phylogenetic tree, and ASV sequences were then bundled into phyloseq objects for further plotting and statistical analysis (phyloseq version 1.24.2). Abundance value transformation was applied to obtain the relative abundance of microbes.

### Statistical analysis and data visualization

Data analysis and visualization was performed using R Studio (version 1.1.456, RStudio, Boston, Massachusetts, USA) and package ggplot2 (version 3.2.1). Statistical analysis was performed using the Wilcoxon test in the stat_compare_means function in the ggpubr package (version 0.2.4), and the Shapiro test, Wilcoxon test, *t* test, and χ^2^ (χ^2^) test in the stats package (version 3.6.1). The p.adjust function in the stats package was used to adjust for multiple comparisons of PERMANOVA results using the Bonferroni method. Statistical significance was defined as a *P* < 0.050.

### Assessment of diversity of the skin and gut microbiota

The mean Shannon diversity index of microbial communities at different skin sites, as well as in the distal gut (stool), was calculated using the unfiltered 16S rRNA gene amplicon sequence data set. Correlation between microbial diversity and age was assessed through fitting of a linear model using the lm function in the stats package.

### Assessment of skin and gut microbiota structure

To calculate the pairwise Bray Curtis dissimilarity between all samples, the 16S rRNA gene amplicon sequence data set was filtered to contain ASVs with a minimum prevalence of 5% across all samples, which corresponded to a minimum read depth of 1000. Principal coordinates analysis (PCoA) in combination with permutational multivariate analysis of variance (PERMANOVA) through the adonis function in the vegan package (version 2.5.0) was used to assess the differences in microbiota structure. The impact of variables – subject ID, study group, sample day, skin site, sex, age, and antibiotics – on variance in microbiota structure was assessed. Furthermore, skin site pairs were ranked by physical distance (assigned ranks: volar forearm-elbow crease = 1, volar forearm-forehead = 2, volar forearm-shin = 2, elbow crease-shin = 2, forehead-shin = 3) and correlation with composition similarity at baseline was assessed through fitting of a linear model using the lm function in the stats package.

### Assessment of impact of emollient therapy on skin and gut microbiota diversity

Linear mixed-effects modelling was used to assess the association between the Shannon diversity index and study group. Because of study design and the concurrent clinical need for administration of antibiotics to treat the combination of SAM and acute diarrhea, we considered the effects of emollient on the microbiota confounded by the effects of antibiotics which are a potent form of microbiota perturbation. Models were fit using the lmer function in the lme4 package (version 1.1.0) and built following the principle of parsimony using a forward-selection approach with variables – study group, sample day, skin site, age, sex, antibiotics, delivery mode, breastfeeding – sequentially added to identify the best fitting model. Nested models were compared with the ANOVA function, which assesses statistically significant improvement after addition of a variable evaluated by the χ^2^ test (*P* = 0.050 was chosen as a cut-off). Additionally, the models were compared and evaluated using the Akaike information criterion (AIC). In the final model for skin diversity, study group, sample day, and body site were included as fixed effects and subject ID as a random effect. The final model for gut diversity included study group, sample day, age in months, and number of antibiotics, as fixed effects and subject ID as a random effect. No absolute correlation values above 0.7 were observed for any model.

### Identification of microbial taxa that distinguish each of the treatment groups

Tree-based sparse discriminant analysis using the treeDa package (version 0.0.4) [29] was performed separately for all four skin sites. This approach incorporates information from the phylogenetic tree, and was used to identify ASVs that differ between study groups during the second half of treatment. The mean relative abundance of all discriminating ASVs across the second half of treatment (days 6-10) was calculated per subject and compared using Wilcoxon test.

## RESULTS

### The skin microbiota of Bangladeshi children with SAM is highly diverse and varies according to physical distance between skin sites

**Figure 1:**
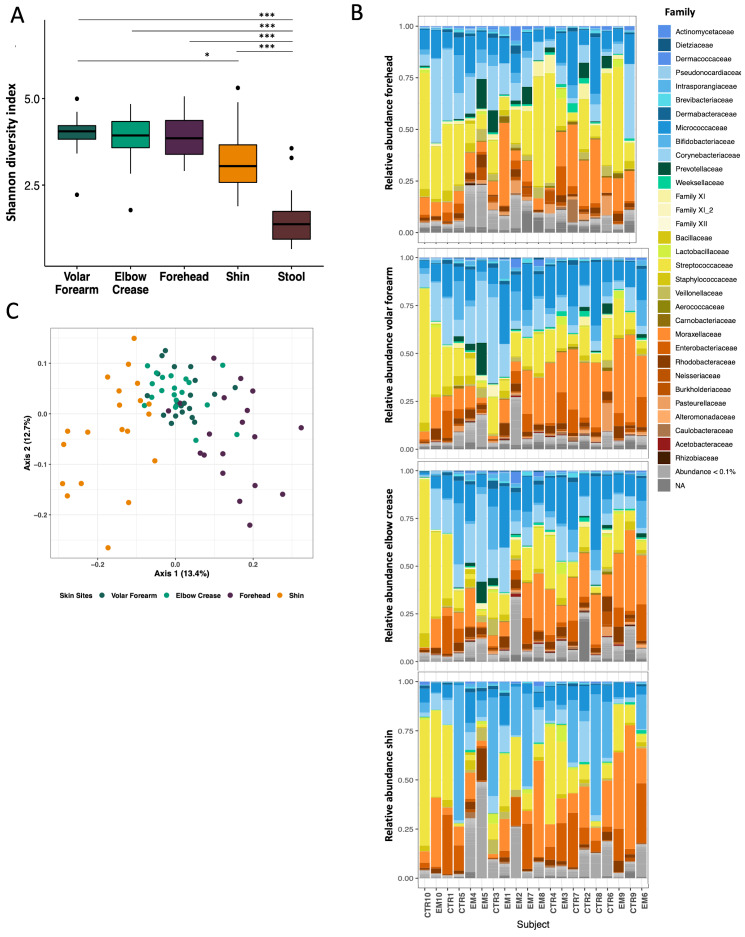
Baseline diversity and structure of the skin microbiota in Bangladeshi children with severe acute malnutrition (SAM) at four different body sites. Bacterial DNA was extracted from skin swabs and fecal samples of 20 Bangladeshi children with SAM at time of enrollment. A) Comparison of mean Shannon diversity between skin sites and stool (Wilcoxon test, *P < 0.05, ***P < 0.001). B) Microbiota structure at skin sites by Family, color-coded by phylum: shades of blue = *Actinobacteria*, shades of green = *Bacteroidetes*, shades of yellow = *Firmicutes*, shades of orange = *Proteobacteria*; light grey = amplicon sequence variants (ASVs) with relative abundance <0.1%, dark grey = ASVs without taxonomic assignment. C) Principal coordinates analysis (PCoA) of the Bray-Curtis dissimilarity for skin samples at baseline. Coordinates were centered per subject. Dark green = volar forearm, light green = elbow crease, orange = shin, purple = forehead.

At baseline, significantly higher microbial diversity was observed at all four skin sites as compared to stool (**Figure 1**, Panel A; Wilcoxon Test, adjusted *P*-value <0.001). Among the skin sites, the shin was less diverse than the volar forearm (**Figure 1**, Panel A; Wilcoxon Test, adjusted *P* = 0.020). We observed a trend in increased microbial diversity with participant age in months, which was most evident at the forehead (Figure S3 in the **Online Supplementary Document**; linear regression, forehead, *P* = 0.035). All four skin sites were dominated by the same four phyla: Actinobacteria, especially the families *Corynebacteriaceae*, *Bifidobacteriaceae*, and *Micrococcaceae*; Proteobacteria, especially *Moraxellaceae* and *Enterobacteriaceae*; Firmicutes, especially *Streptococcaceae*; and Bacteroidetes, represented by *Prevotellaceae* and *Weeksellaceae* (**Figure 1**, Panel B). The dominant families were similar across different skin sites within the same child.

PCoA of the pairwise Bray-Curtis dissimilarity between all samples showed clustering by skin site, with overlap of volar forearm and elbow crease samples (**Figure 1**, Panel C). We detected a strong individual signature as well as impact of body site, age and sex on microbiota structure (Table S2 in the **Online Supplementary Document**; PERMANOVA: subject ID R^2^ = 0.527, adj. *P* = 0.004; body site R^2^ = 0.091, adj. *P* = 0.004; age in months, R^2^ = 0.059, adj. *P* = 0.004; sex R^2^ = 0.031, adj. *P* = 0.004).

**Figure 2:**
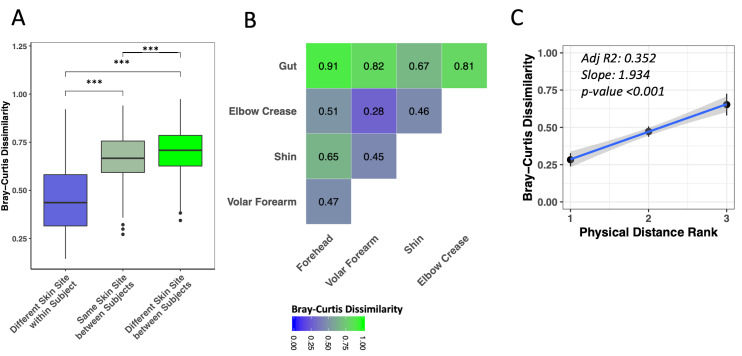
Similarity of microbiota structure at different skin sites within and between Bangladeshi children with severe acute malnutrition (SAM). A) Comparison of mean Bray-Curtis dissimilarity between baseline samples from different and the same skin sites within and between children. B) Comparison of mean Bray-Curtis dissimilarity between baseline samples from different skin sites of the same child. C) Linear regression of mean Bray-Curtis dissimilarity against physical distance between body sites (assigned ranks: volar forearm-elbow crease = 1, volar forearm-forehead = 2, volar forearm-shin = 2, elbow crease-shin = 2, forehead-shin = 3). Grey shaded area represents the 95% confidence interval (CI). *P* values are displayed for between-sample comparisons (Wilcoxon test, ***P < 0.001).

Different skin sites on the same child were significantly more similar to each other than were the same skin sites among different children (**Figure 2**, Panel A, Wilcoxon test, adj. *P* < 0.001). Nonetheless, the same skin sites on different children were more similar to each other than were different skin sites on different children (**Figure 2**, Panel A, Wilcoxon test, adj. *P* < 0.001). Among skin sites on the same child, the volar forearm and elbow crease were most similar to each other (**Figure 2**, Panel B; Figure S4 in the **Online Supplementary Document**, adj. *P* < 0.01 to all other pairs), while shin and forehead were most distinct (**Figure 2**, Panel B; Figure S4 in the **Online Supplementary Document**, adj. *P* < 0.05 to all other pairs, except forehead-elbow crease adj. *P* = 0.195). We observed evidence for correlation of ranked physical distance with compositional similarity (**Figure 2**, Panel C, linear regression *P* < 0.001).

### Short-term emollient therapy had subtle effects on skin microbiota structure

**Figure 3:**
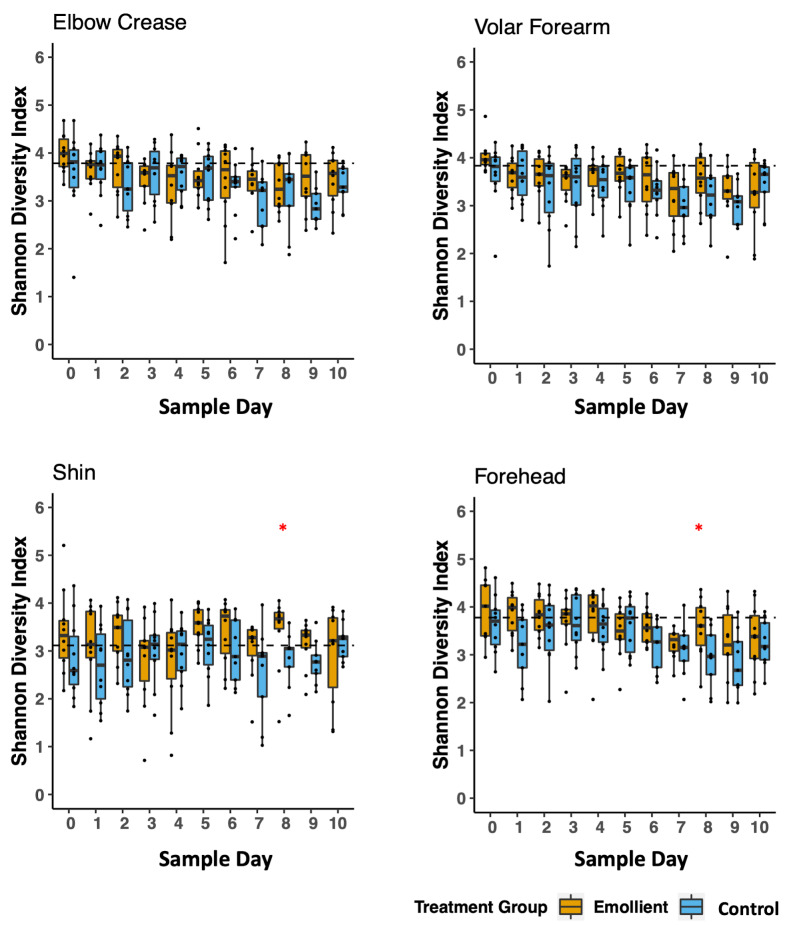
Temporal trends in skin microbiota diversity during the study period per skin site and treatment group in Bangladeshi children with severe acute malnutrition (SAM) undergoing topical emollient therapy. Mean Shannon diversity index was calculated per sample day, skin site, and study group, and compared using the Wilcoxon test (*P < 0.05). Blue bars = control group, yellow bars = emollient group.

There was a tendency for a higher mean Shannon diversity index in the emollient group than in the control group in the second half of treatment. This observation was evident on the forehead (Wilcoxon test, *P* = 0.035) and on the shin (Wilcoxon test, *P* = 0.022) on study day 8 (**Figure 3**). Through linear mixed-effects modelling including the variables, study group, sample day, and body site as fixed effects and subject ID as a random effect, we noted a decrease in Shannon diversity index per sample day (Table S3 in the **Online Supplementary Document**, coeff = -0.037, *P* < 0.001), which was less evident in the emollient group (coeff = 0.198, *P* = 0.172).

**Figure 4:**
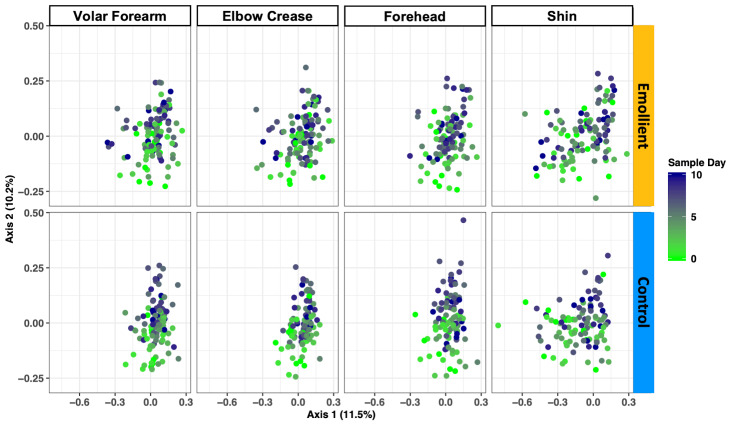
Skin microbiota structure during the study period per skin site and treatment group in Bangladeshi children with severe acute malnutrition (SAM) undergoing topical emollient therapy. Principal coordinates analysis (PCoA) of the Bray-Curtis dissimilarity was performed per skin site and treatment group. Color gradient: sample days, light green = early days, purple = later days.

PCoA of the Bray-Curtis dissimilarity between samples showed a change in structure along axis 2 by sample day at all four skin sites and in both study groups (**Figure 4**). We observed a difference in microbiota structure between study groups (PERMANOVA: R^2^ = 0.018, adj. *P* = 0.005), as well as an impact of sample day (R^2^ = 0.024, adj. *P* = 0.005), skin site (R^2^ = 0.030, adj. *P* = 0.005), sex (R^2^ = 0.026, adj. *P* = 0.005), and age (R^2^ = 0.03, adj. *P* = 0.005) (Table S4 in the **Online Supplementary Document**).

### Emollient therapy increased abundance of *Prevotellaceae* at several skin sites

The family with the greatest number of ASVs significantly associated with emollient treatment was *Prevotellaceae*. We identified an average number of 54 (50-58) discriminating ASVs affiliated with the genus *Prevotella* at the elbow crease, shin and forehead, of which 24 were common to all three skin sites (Figures S5-S8, Table S5 in the **Online Supplementary Document**). Relatively few ASVs were associated with the control group, and many of them were common across different skin sites, including the same four ASVs affiliated with *Stenotrophomonas* at the volar forearm, elbow crease and forehead.

**Figure 5:**
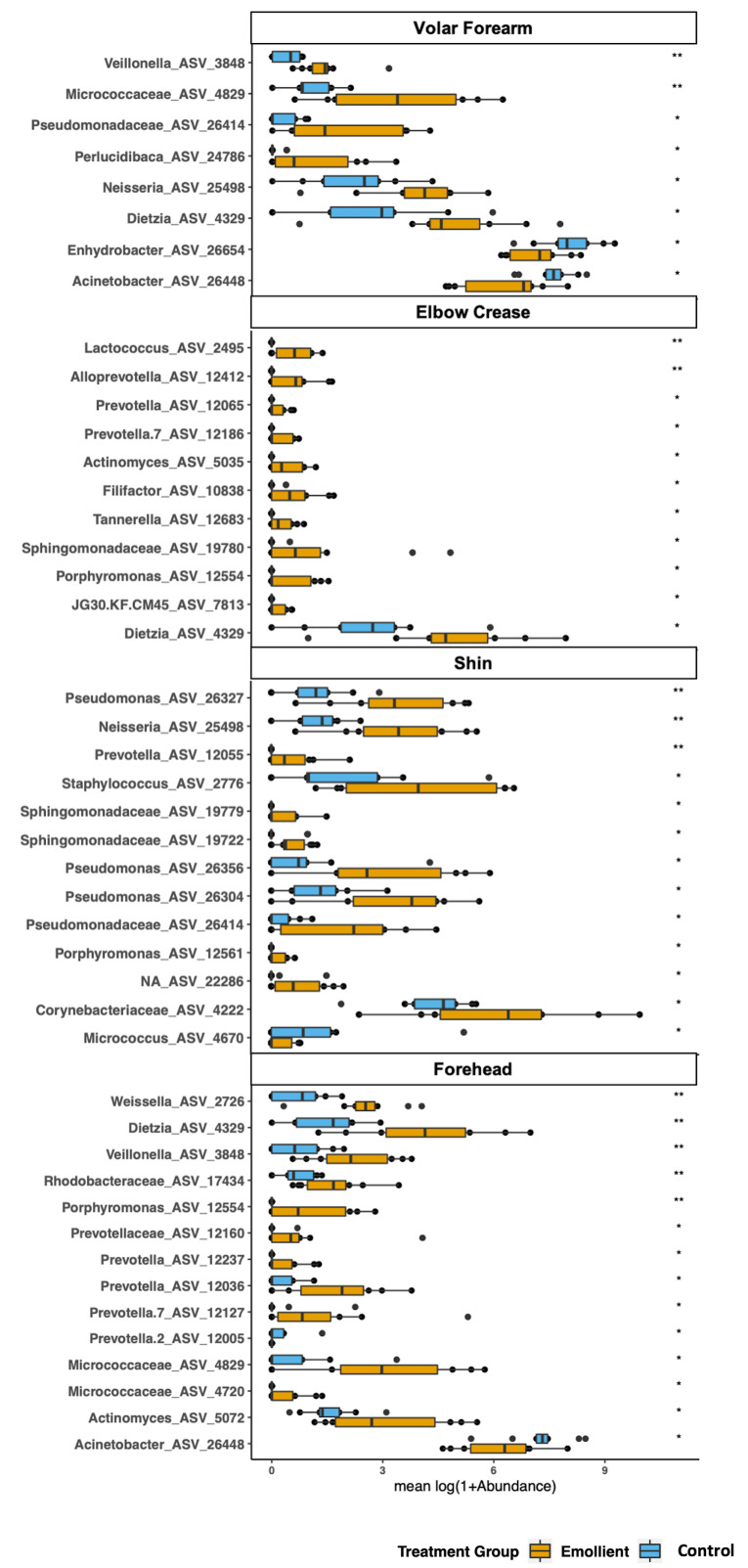
Bacterial taxa with significant differences in abundance between treatment groups during the second half of treatment in Bangladeshi children with severe acute malnutrition (SAM) undergoing topical emollient therapy. The mean relative abundance of amplicon sequence variants (ASVs) identified by linear discriminant analysis as differentiating between the treatment groups on days 6-10 was calculated per subject and skin site. Means were compared using the Wilcoxon test. **P < 0.01, *P < 0.05. The *P* values presented here were not corrected for multiple comparisons. ASVs are presented by skin site. Blue bars = control group, yellow bars = emollient group.

We identified 34 ASVs with significantly higher abundance in the emollient group and four ASVs with significantly higher abundance in the control group (**Figure 5**). Furthermore, 45 ASVs increased in mean relative abundance from baseline in the emollient group, and four ASVs decreased; while 13 ASVs increased in mean relative abundance from baseline in the control group, and 10 ASVs decreased (Figure S9 in the **Online Supplementary Document**).

**Figure 6:**
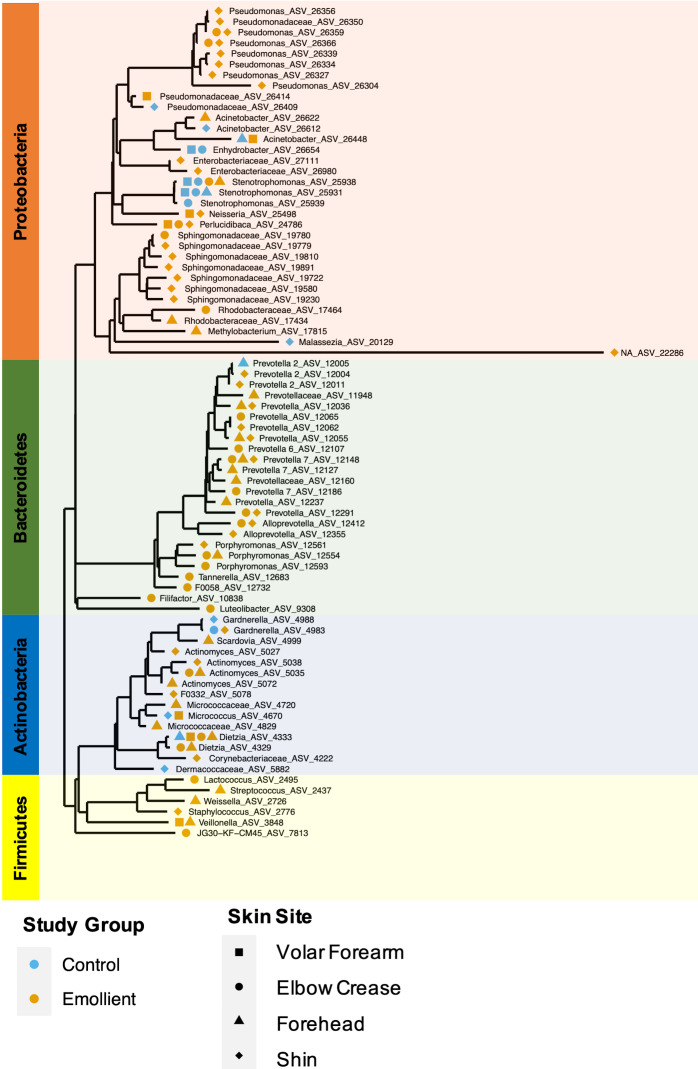
Phylogenetic tree of amplicon sequence variants (ASVs) that discriminated between treatment groups in Bangladeshi children with severe acute malnutrition (SAM) undergoing topical emollient therapy. Blue symbol = control group, yellow symbol = emollient group; square = volar forearm, circle = elbow crease, triangle = forehead, diamond = shin. Background color indicates phylum (yellow = *Firmicutes*, blue = *Actinobacteria*, green = *Bacteroidetes*, orange = *Proteobacteria*).

Overall, the phylogenetic groups whose abundance was most impacted by emollient therapy were *Prevotellaceae* with 16 ASVs associated with four different genus-level taxa (*Prevotella*, Prevotella 2, Prevotella 7 and *Alloprevotella*), followed by *Pseudomonadaceae* and *Sphingomonadaceae,* among others (**Figure 6**). Microbes with abundance most impacted in the control group were affiliated with *Stenotrophomonas*, *Moraxellaceae*, and *Gardnerella*, among others. Microbes affiliated with the same genus or family were found to exhibit similar behavior across two or more distant skin sites.

### Short term emollient therapy does not affect the gut microbiota of children with SAM

At baseline, the gut microbiota of the majority of children with SAM was dominated by *Pseudomonadaceae*, with the microbiota of the others by either *Streptococcaceae*, *Lactobacillaceae*, *Bifidobacteriaceae,* or *Prevotellaceae* (Figure S10, Panel A in the **Online Supplementary Document**). Diagnoses in three children (EM7, CTR3, and CTR6) of Campylobacter infection and in one child (CTR9) of cholera, based on positive stool cultures for these pathogens, were supported by the data from 16S rRNA gene amplicon libraries in which *Campylobacteraceae* or *Vibrionaceae* ASV sequences were prominently featured, respectively. Additionally, four other participants (EM1, EM6, CTR2, and CTR8) had substantial numbers of *Campylobacteraceae* 16S rRNA sequence reads, but had not been diagnosed with Campylobacter infection in the clinical microbiology laboratory. At baseline, we observed a weak but non-significant correlation of gut microbiota diversity with age in months as a single factor in our cohort (Figure S10, Panel B; and Table S5 in the **Online Supplementary Document**, linear regression, *P* = 0.061).

**Figure 7:**
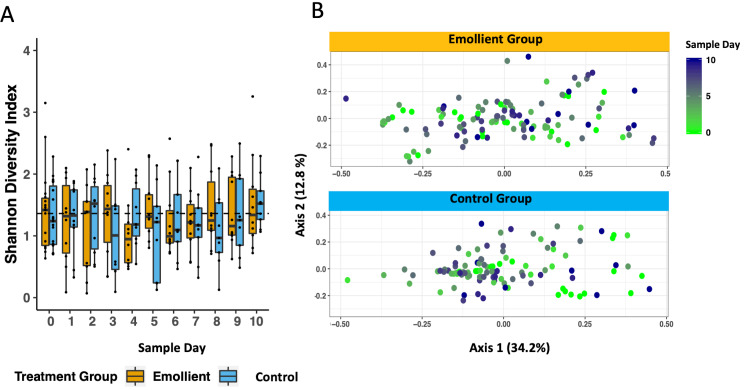
Gut microbiota diversity and structure during the study period in Bangladeshi children with severe acute malnutrition (SAM) undergoing topical emollient therapy. A) Mean Shannon diversity was calculated for each day and compared between study groups. B) Principal coordinates analysis (PCoA) was performed on the Bray-Curtis dissimilarity between stool samples. Color gradient: sample days, light green = early days, purple = later days.

We noted no decline in gut microbiota diversity over the study period (**Figure 7**, Panel A). Through linear mixed-effects modelling including the variables, study group, sample day, age in months, and number of antibiotics as fixed effects, and subject ID as a random effect, we detected an impact of age and to a lesser extent, the number of different antibiotics a participant had received on the Shannon diversity index, but no impact of emollient therapy (Table S6 in the **Online Supplementary Document**, **Figure 6**; age in months: coeff = 0.091, *P* < 0.001; number of antibiotics: coeff = -0.285, *P* = 0.054; emollient: coeff = 0.044, *P* = 0.748).

PCoA of the Bray-Curtis dissimilarity between samples indicated an association between gut microbiota structure and sample day, age in months, a trend towards impact of study group, but not sex or number of antibiotics (Table S7; PERMANOVA: sample day: R^2 =^ 0.010, adj. *P* = 0.045; age in months: R^2 =^ 0.103, adj. *P* = 0.045; study group: R^2 =^ 0.022, adj. *P* = 0.060).

## DISCUSSION

### The skin microbiota of Bangladeshi children with SAM differs from that of healthy Western Caucasian children

Our study is one of the first to describe the skin microbiota in children from a low- or middle-income country (LMIC). We found that the skin microbiota structure of Bangladeshi children with SAM aged 2-24 months living in urban Dhaka was highly diverse, even more so than their gut microbiota. Their skin microbiota at four different sites was dominated by members of the genera *Bifidobacterium*, *Corynebacterium*, and *Micrococcus* within the phylum *Actinobacteria*; the genus *Streptococcus* within the phylum *Firmicutes*; and the genus *Acinetobacter* and *Enhydrobacter* within the phylum *Proteobacteria*. In contrast, the skin microbiota of healthy children of the same ages from high income countries are dominated by members of the genus *Propionibacterium* within the phylum *Actinobacteria*; and members of the genus Staphylococcus within the phylum *Firmicutes* [14]. In healthy Western children, skin microbiota diversity increases with age [14]; we also observed a trend towards increased diversity with age, especially at the forehead.

We found that the skin microbiota was specific to the individual, but that skin site-specific patterns were less prominent than reported in studies of adults from the US [13,30]. Similarities in skin microbiota structure did not appear to be associated with skin habitat type (dry, moist, or sebaceous) as has been described in US adults, but rather with physical distance between skin sites, although our study was not designed to test this association in a rigorous manner.

Differences in sanitation, lifestyle, and the environmental microbiome of urban Dhaka vs Western settings may lead to higher degrees of microbial exposure and diversity overall and less distinct microbiota structures at different skin sites. Recent work by Lehtimäki et al. highlighted the importance of the living environment in shaping the skin microbiota of Finnish children living either in an urban or rural environment, especially in toddlers, as well as the relationship to development of allergic disease [31]. Those differences in composition across distinct populations with varying lifestyles may have clinical relevance.

### Emollient therapy affects the skin microbiota both directly and indirectly

Our results indicated that SSO has an effect on the skin microbiota at sites receiving direct oil massage, as well as distant untreated skin sites (ie, the forehead). Overall, the emollient group showed a trend towards higher skin microbiota diversity later in the course of treatment, which may be a beneficial effect, since low diversity or dominance of one taxon in the skin microbiota has been associated with disease, such as atopic dermatitis [32] or psoriasis [33]. Moreover, emollient therapy had specific effects on skin microbiota structure, including consistent, significant changes in abundance of multiple ASVs within the same genus across multiple skin sites. These observations suggest a selective effect, rather than just passive retention of random species on the skin. Moreover, our data showed a shared effect among similar taxa at different skin sites receiving emollient, as well as at a distant site without direct treatment.

There are several potential modes of action by which emollient might affect the microbiota. As a result of an improvement in skin barrier function, hydration levels in the skin would be expected to increase and natural lipid composition change, hence favoring growth by certain microbial species. The skin produces an array of lipids, not found elsewhere in the body, which in turn can be utilised and metabolised by certain microbes such as *Propionibacterium* spp [34,35]. Interestingly, *Propionibacterium* was at extremely low abundance in our samples, even at sebaceous sites such as the forehead, and did not increase with emollient treatment. Alternatively, SSO itself could act as a nutrient source for members of the skin microbiota.

Fatty acids derived from sebaceous triglycerides have been shown to possess antimicrobial activity [36, 37]. It is possible that emollient alters skin pH, which in turn is linked to production of cationic antimicrobial peptides by keratinocytes. Both could therefore impact the skin microbiota composition. Another mechanism may be immune-mediated and elicited by SSO or by microbes or their products. SSO is mostly comprised of triglycerides such as linoleic acid and oleic acid, with smaller proportions of palmitic and stearic acid. Peroxisome proliferator-activated receptors (PPARs) are able to sense fatty acids, such as linoleic and stearic acid, and regulate inflammatory responses [38]. Furthermore, short chain fatty acids produced by the skin microbiota can influence cytokine expression through inhibition of histone deacetylases and thereby regulate tolerance and inflammation in the skin [39].

### Why are *Prevotella* affected and what could that mean for health?

We observed a strong impact of SSO on four different genera of *Prevotellaceae*, resulting in significant increases in abundance across several skin sites, especially during the later days of treatment. *Prevotellaceae* are anaerobic and may thrive in skin habitats with lower oxygen concentrations such as the sebaceous glands and hair follicles, which may be rendered even more anaerobic by SSO.

Capone et al reported a low percentage of *Prevotella* on the skin of healthy Western infants [14], but little is known about *Prevotellaceae* in the skin ecosystem or their role in skin health or disease. In the gut, increased *Prevotella* abundance has been linked to augmented T helper type-17 (Th17) activation [40], but nothing is known about their potential to prime the Th17 immune circuit in the skin. Th17 cells and their associated cytokines – IL-17 and IL-22 – provide signaling to epithelial cells to enhance anti-microbial defense through the induction of antimicrobial peptides [41], and improve epithelial barrier integrity, through induction of keratinocyte proliferation and tissue regeneration after injury [42]. Such effects could prove beneficial in the recovery from SAM by preventing infectious agents from invading through the skin [43].

### Gut microbiota and skin-gut-axis

A recent study on the skin microbiota development of infants in a hospital setting showed direct exchange of microbes across body sites – including between skin and gut – using bacterial source tracking [44]. Changes in skin microbiota structure through topical emollient therapy could therefore be expected to impact the gut as well. The gut microbiota of children with SAM has been found to be immature, a state not easily corrected by nutritional intervention alone [45,46]. We observed low diversity in the gut microbiota of children in our cohort at baseline, which together with acute diarrhea and antibiotic administration may have affected our ability to detect subtle changes exerted by SSO, especially during a relatively brief study period; however, the impact of emollient therapy on gut microbiota composition over time warrants further investigation. The connections between the skin and gut, as well as impacts of topical emollient therapy on gut microbiota and barrier function remain an exciting area of future exploration.

### Limitations and future work

One must keep in mind that all of the children in our cohort suffered from SAM when comparing our results to those of healthy children, and that nutritional status may have important effects on skin microbiota. Since our study population suffered from acute diarrheal disease in combination with SAM, all children received a standard regimen of antibiotics concurrent with emollient therapy. Antibiotics are known to disturb microbiotas and are expected to have impeded our ability to identify changes caused by emollient.

Our goal was to explore the impact of SSO on microbiota structure and our findings need to be confirmed in larger studies, with longer treatment and sampling periods, without the confounding effects of antibiotics, and with the means for clinical follow-up. Significance values for comparisons of mean abundances were not corrected for multiple comparisons in the analysis shown in **Figure 5** since a major goal of this work was hypothesis generation.

Community-based studies should be undertaken to understand better the interplay of oil massage practice and the living environment on the skin microbiota, as well as the role of transfer of microbes by skin-to-skin contact with the caregiver [47].

In addition, new technologies may yield further insights into the mechanisms underlying the impact of SSO on the microbiota as well as on skin and gut barriers. Work by Bouslimani et al*,* mapping the molecular composition of human skin found that the chemical milieu is strongly defined by daily applications of hygiene products, which can still be detected several days after use [48]. Future analyses using mass spectrometry may elucidate the metabolism of SSO-derived fatty acids by the skin microbiota. Furthermore, metagenomic sequencing of *Prevotellaceae* strains may shed light on their capacity to metabolize EFAs and their downstream products. Connections between the microbiota and the skin immune system are beginning to be unraveled and warrant further exploration in the setting of emollient therapy. Massage practices themselves may influence the host immune system and thereby microbiota composition.

## CONCLUSIONS

Clinical trials have demonstrated beneficial effects of emollient therapy with SSO on skin barrier function, as well as protection from nosocomial infections leading to decreased morbidity and mortality in very preterm infants in LMICs [6,7].

Our study adds an important layer to these findings by indicating that emollient therapy with SSO can affect skin microbiota diversity and structure in children with SAM. Strong evidence points towards a time window of gut microbiota maturation and heightened susceptibility to disturbance very early in life [49]. The same may be true for the skin microbiota, but this remains to be confirmed. This critical time window offers opportunities for therapeutic interventions with the capacity to shift the microbiota in a manner that promotes health later in life [46,50]. Infant massage with natural plant oils is a long-standing, widespread traditional practice in many LMICs in the Mediterranean region, Africa and Asia, which might be leveraged by promoting the use of more beneficial, non-traditional oils such as SSO [51]. Studies of the microbiota in children in LMICs and its relationship to health and disease are still relatively rare, but deserve special attention.

## Additional material

Online Supplementary Document
