# Supplementary material for: Topical emollient therapy with sunflower seed oil alters the skin microbiota of young children with severe acute malnutrition in Bangladesh: A randomised, controlled study
Source: J Glob Health. 2021 Jul 17;11:04047. doi: 10.7189/jogh.11.04047 (PMC8325932; doi:10.7189/jogh.11.04047)
Supplement: Online Supplementary Document [file jogh-11-04047-s001.pdf]

Online Supplementary Document

Topical emollient therapy with sunflower seed oil alters the skin microbiota of young children with severe acute malnutrition in Bangladesh: a randomised, controlled study

Natalie Fischer, Gary L Darmstadt, KM Shahunja, Jonathan M Crowther, Lindsay Kendall, Rachel A Gibson, Tahmeed Ahmed, David A Relman

Appendix S1. Supplementary Methods

Study population. All children in the study received SAM routine standard-of-care, which includes rehydration, nutrition and antibiotics (1). Oil massage was performed by trained nurses, who washed their hands with soap and water prior to oil application and performed gentle massage with bare hands. Oil was applied starting from the hands, then from front to the back of the body, followed by the legs and finally the buttock area. The buttock area was massaged outwards to inwards to prevent spreading of enteric bacteria from close to the anal area.

Sample collection. Skin samples were obtained by swabbing a 2x2 cm area of the skin with a sterile swab moistened with sterile sample buffer (0.15 M NaCl, 0.1% Tween 20). Samples of collection buffer, as well as empty swabs were retained as negative controls. All samples were frozen immediately at -80°C, and later shipped to Stanford University on dry ice for storage and subsequent analysis.

16S rRNA gene amplicon sequencing. DNA was extracted from ~200 mg of stool using the QIAGEN DNeasy PowerSoil HTP 96 Kit (Cat #12955-4) following the manufacturer's instructions, including a 2 x 10-minute bead-beating step using the Retsch 96 Well Plate Shaker at speed 20. DNA from skin swabs was isolated using the QIAamp BiOstic Bacteremia DNA Kit (Cat#: 12240-50) according to the manufacturer's protocol, including a 15 min incubation at 70°C followed by bead beating with a MPBio FastPrep-24™ 5G Homogenizer (Cat#: SKU

116005500) for 60s at 6m/s. Empty collection tubes, unused swabs, as well as collection buffer were extracted and amplified in the same way to serve as negative controls.

The V4 region of the bacterial 16S rRNA gene was amplified by PCR using barcoded Illumina forward primer 515F (5′-GTGCCAGCAGCCGCGGTAA-3′) with an error-correcting barcode and reverse primer 806R (5′-GGACTACCAGGGTATCTAAT-3′) (2). Amplicons were purified using the Qiagen UltraClean 96 PCR Cleanup kit (Cat#: 12596-4) and quantified using the Quant-iT™ dsDNA Assay Kit (Thermo Fisher Scientific, Cat#: Q33120) and pooled in equimolar concentrations. The amplicon pool was then concentrated using a DNA Clean & Concentrator™ column (Zymo Research, Cat# D4031) and sequenced with a 2x250nt protocol on two lanes of a HiSeq 2500, using a HiSeq Rapid SBS sequencing kit version 2. A total of 391,682,822 high quality reads were generated from the 232 stool samples and 859 skin swabs (~324,570 reads/sample (sd 33,160 reads)). Fastq files were demultiplexed with the bcl2fastq v2.20 (Illumina).

Data processing and ecological statistics. Raw sequencing reads were demultiplexed using the QIIME command split_libraries_fastq.py (QIIME version 1.9.1) and then quality trimmed using the DADA2 pipeline (dada2 version 1.1.1) in R (R version 3.2.4) (3). Briefly the first ten nucleotides were trimmed from the left side and all reads were quality filtered with settings maxN=0, maxEE=2, truncQ=11. Following quality trimming, amplicon sequence variants (ASVs) were inferred using the DADA2 pipeline which resulted in 32.299 ASVs identified in 1214 samples (including negative controls). Taxonomy was assigned to each ASV using the IDTAXA (4) classifier and the SILVA 16S rRNA database [SILVA SSU r132 (March 2018)], and a phylogenetic tree was built from the ASVs using the QIIME2 function q2-fragment-insertion (5). The ASV table, patient sample data, taxonomy assignments, phylogenetic tree, and ASV sequences were then bundled into phyloseq objects for further plotting and statistical analysis (phyloseq version 1.24.2) (6). The decontam package was used separately on skin and stool samples to remove contaminants based on prevalence in negative controls versus samples (7). Mitochondria sequences were removed from the dataset.

Sparse discriminant analysis from the treeDa package (version 0.0.4) (8), which uses information from the phylogenetic tree, was used to identify taxa that differentiated between treatment groups. Only ASVs present in at least 25% of participants as well as sample days 6-10

were included in the analysis per body site, resulting in 1305 taxa for the volar forearm, 1307 taxa for the elbow crease, 1080 taxa for the shin, and 1310 taxa for the forehead. After cross validation, samples from the volar forearm were differentiated by 16 predictors, which corresponded to 59 leaves on the tree; samples from the elbow crease were differentiated by 22 predictors, which corresponded to 174 leaves on the tree; samples from the shin were differentiated by 29 predictors, which corresponded to 201 leaves on the tree; and samples from the forehead were differentiated by 22 predictors, which corresponded to 147 leaves on the tree.

Product description of refined sunflower seed oil.

We used cold pressed linoleic acid-rich (48-74%), low oleic acid (14-39%) containing SSO donated from a commercial supplier (Cargill Refined Oils, Europe) (see Table below for a full specification of the SSO). The oil was stored at -20°C in aliquots, defrosted and allowed to reach room temperature before use. After defrosting, the oil was maintained at room temperature for up to 7 days after which it was discarded and replaced with fresh oil.

TECHNICAL SPECIFICATION*				
		**Min**	**Max**	***Reference method*** †
**Sensory:**				
Taste	-	bland		*Cargill internal method*
Appearance at room temperature	-	clear		*Cargill internal method*
**Chemical:**				
Free Fatty Acid, as oleic	%	-	0.10	*EN-ISO 660:2009*
Peroxide Value, at bottling	mq/kg	-	2.0	*ISO 3960:2007*
Moisture Content	%	-	0.10	*ISO 8534:2007*
Colour Lovibond 5.25”	Red	-	2.0	*ISO 15305:1998*
**Fatty Acid Composition:**				*EN-ISO 5509:2000 & EN-ISO 5508:1995*
C16:0	%	5.0	7.6	
C18:0	%	2.7	6.5	
C18:1 (total)	%	14.0	39.4	
C18:2 (total)	%	48.3	74.0	
C18:3 (total)	%	-	0.5	
Trans fatty acids (total)	%	-	2.0	

* Analyses are performed by refineries/ suppliers before reception of the oils in the bottling plant

† *Cargill reserves the right to use internal analytical method that is in compliance with the International Reference Method*

Supplementary References

1. Ahmed T, Ali M, Ullah MM, *et al.* Mortality in severely malnourished children with diarrhoea and use of a standardised management protocol. Lancet 1999;353:1919–1922.

2. Caporaso JG, Kuczynski J, Stombaugh J, *et al.* QIIME allows analysis of high-throughput community sequencing data. Nat Meth 2010;7:335–336.

3. Callahan BJ, McMurdie PJ, Rosen MJ, *et al.* DADA2: High-resolution sample inference from Illumina amplicon data. Nat Meth 2016;13:581–583.

4. Murali A, Bhargava A, Wright ES. IDTAXA: a novel approach for accurate taxonomic classification of microbiome sequences. Microbiome 2018;6:140.

5. Janssen S, McDonald D, Gonzalez A, *et al.* Phylogenetic Placement of Exact Amplicon Sequences Improves Associations with Clinical Information. mSystems 2018;3:581–14.

6. McMurdie PJ, Holmes S. phyloseq: an R package for reproducible interactive analysis and graphics of microbiome census data. PLoS ONE 2013;8:e61217.

7. Davis NM, Proctor DM, Holmes SP, *et al.* Simple statistical identification and removal of contaminant sequences in marker-gene and metagenomics data. Microbiome 2018;6:226.

8. Fukuyama J, Rumker L, Sankaran K, *et al.* Multidomain analyses of a longitudinal human microbiome intestinal cleanout perturbation experiment. PLoS Comput. Biol. 2017;13:e1005706–29.

9. Darmstadt GL, Ahmed S, Ahmed ANU, *et al.* Mechanism for Prevention of Infection in Preterm Neonates by Topical Emollients. The Pediatric Infectious Disease Journal 2014;33:1124–1127.

10. Darmstadt GL, Mao-Qiang M, Chi E, *et al.* Impact of topical oils on the skin barrier: possible implications for neonatal health in developing countries. Acta Paediatr. 2002;91:546–554.

11. Chiou YB, Blume-Peytavi U. Stratum corneum maturation. A review of neonatal skin function. Skin Pharmacol Physiol 2004;17:57–66.

12. Gensollen T, Iyer SS, Kasper DL, *et al.* How colonization by microbiota in early life shapes the immune system. Science 2016;352:539–544.

Supplementary Tables

Table S1. Baseline characteristics and antibiotic treatment of study participants. 20 children with severe acute malnutrition (SAM) were randomized into either the emollient treatment group or the control group. There were no statistical differences in baseline characteristics or in use of antibiotics (t-test for age, TEWL; Wilcoxon test for WLZ, days of gentamicin, days of amoxicillin, days of ampicillin, chi2-test for sex, delivery mode, breastfeeding). WLZ = weight-for-length z score, TEWL = trans-epidermal water loss, C-section = cesarian section.

	Emollient (n=10)	Control (n=10)
Male sex, n (%)	7 (70%)	7 (70%)
Age range, months	2.3-18.0	2.3-13.2
Age, months, mean (± sd)	8.1 (± 4.9)	7.8 (± 3.5)
WLZ at admission, mean (± sd)	-3.4 (± 0.34)	-3.5 (± 0.52)
TEWL at admission, mean (± sd)	12.6 (± 3.2)	13.9 (± 4.3)
Days of gentamicin after admission, mean (± sd)	8.1 (± 0.74)	7.8 (± 0.79)
Days of amoxicillin after admission, mean (± sd)	5.5 (± 0.76)	5.25 (± 1.39)
Days of ampicillin after admission, mean (± sd)	3.5 (± 1.3)	3.7 (± 0.82)
Delivery mode, C-section, n (%)	3 (30%)	1 (10%)
Breastfeeding at admission, n (%)	6 (60%)	9 (90%)
Exclusively breastfeeding at admission, n (%)	5 (50%)	7 (70%)

Table S2. Results of PERMANOVA (Adonis) for the impact of body site, subject ID, age, and sex on skin microbiome structure in Bangladeshi children with severe acute malnutrition (SAM) at baseline. See Figure 1, panel C.

**Model**	**adonis(ps_bray ~ age_m + sex + Body_Site + Subject_ID , data = sampledf, permutations = 1000)**					
	**Df**	**SumsOfSqs**	**MeanSqs**	**F.Model**	**R^2**	**adjusted *P* value**
**Subject ID**	17	10.3206	0.60709	5.9374	0.52675	0.004 **
**Body Site**	3	1.7823	0.59411	5.8104	0.09097	0.004 **
**Age (month)**	1	1.1638	1.16376	11.3815	0.05940	0.004 **
**Sex**	1	0.6005	0.60045	5.8724	0.03065	0.004 **
**Residuals**	56	5.7260	0.10225	0.29224		
**Total**	78	19.5931	1.00000			

Abbreviations: Df = degrees of freedom; SumsOfSqs = sequential sums of squares; MeanSqs = mean squares; F.Model = F statistics; R^2 = partial R-squared.

Table S3. Results of linear mixed effect models for Shannon diversity index and association of bacterial skin community diversity with study group in Bangladeshi children with severe acute malnutrition (SAM) undergoing topical emollient therapy.

**Model**	**lmer(Shannon ~ study_group + Sample_Day + Body_Site + (1|Subject_ID), data = df)**			
**Random effects:**				
**Groups**	**Name**	**Variance**	**Std.Dev.**	
**Subject ID**	Intercept	0.09007	0.3001	
**Residual**		0.30897	0.5559	
**Fixed effects:**				
	**Estimate**	**Std. Error**	**t value**	**P value**
**(Intercept)**	3.736725	0.108190	34.538	
**Emollient group**	0.198371	0.139581	1.421	0.172
**Sample Day**	-0.037178	0.006061	-6.134	<0.001***
**Volar Forearm**	-0.018875	0.053743	-0.351	0.726
**Elbow Crease**	-0.030304	0.053741	-0.564	0.573
**Shin**	-0.437851	0.053741	-8.147	<0.001***
AIC: 1515.783				

Abbreviations: Std.Dev. = standard deviation; Std. Error = standard error; AIC = Akaike information criterion.

Table S4. Results of PERMANOVA (Adonis) for the association of skin microbiota structure with study group in Bangladeshi children with severe acute malnutrition (SAM) undergoing topical emollient therapy.

**Model**	**adonis(ps_bray ~ study_group + Sample_Day + Body_Site + sex + age_m , data = sampledf, permutations = 1000, strata=sampledf$Subject_ID)**					
	**Df**	**SumsOfSqs**	**MeanSqs**	**F.Model**	**R^2**	**adjusted P value**
**Study Group**	1	3.806	3.8064	17.0852	0.01757	0.005 **
**Sample Day**	1	5.232	5.2318	23.4831	0.02415	0.005 **
**Body Site**	3	6.464	2.1548	9.6717	0.02983	0.005 **
**Sex**	1	5.544	5.5444	24.8863	0.02559	0.005 **
**Age (months)**	1	6.259	6.2589	28.0933	0.02889	0.005 **
**Residuals**	850	189.371	0.2228	0.87398		
**Total**	857	216.677	1.00000			

Abbreviations: Df = degrees of freedom; SumsOfSqs = sequential sums of squares; MeanSqs = mean squares; F.Model = F statistics; R^2 = partial R-squared.

Table S5. Results of linear regression for the relationship of Shannon diversity index of the gut microbiota of Bangladeshi children with severe acute malnutrition (SAM) at baseline against age in months. See Figure S10, panel B.

**Model**	**lm(mean ~ age months, data = df)**			
**Coefficients:**				
	**Estimate**	**Std. Error**	**t value**	***P* value**
**Intercept**	0.92709	0.25274	3.668	
**Age months**	0.05653	0.02827	2.000	0.061
Residual standard error: 0.5095 on 18 degrees of freedom<br>Multiple R-squared: 0.1818<br>Adjusted R-squared: 0.1363<br>F-statistic: 3.999 on 1 and 18 DF<br>*P* value: 0.061				

Abbreviations: Std. Error = standard error, DF= degrees of freedom.

Table S6. Results of linear mixed effect models for the association between study group and Shannon diversity index for gut bacterial communities in Bangladeshi children with severe acute malnutrition (SAM) undergoing topical emollient therapy.

**Model**	**lmer(Shannon ~ study_group + Sample_Day + age_m + nr antibiotics taken + (1|Subject_ID), data = df)**			
**Random effects:**				
**Groups**	**Name**	**Variance**	**Std.Dev.**	
**Subject ID**	(Intercept)	0.0770	0.2775	
**Residual**		0.1573	0.3966	
**Fixed effects:**				
	**Estimate**	**Std. Error**	**t value**	**P value**
**(Intercept)**	0.659011	0.175389	3.757	
**Emollient**	0.044377	0.135679	0.327	0.748
**Sample Day**	0.011204	0.007962	1.407	0.161
**Age (months)**	0.091388	0.016858	5.421	<0.001***
**Nr. of ABX**	-0.284553	0.136722	-2.081	0.054
AIC: 297.1339				

Abbreviations: Std.Dev. = standard deviation; Std. Error = standard error; Nr. of ABX = Number of different antibiotics; AIC = Akaike information criterion.

Table S7. Results of PERMANOVA (Adonis) for the association between gut microbiota structure and study group in Bangladeshi children with severe acute malnutrition (SAM) undergoing topical emollient therapy.

**Model:**	**adonis(ps_bray ~ study_group + Sample_Day + age_m + sex + days_of_abx, data = sampledf, permutations = 1000, strata=sampledf$Subject_ID)**					
	**Df**	**SumsOfSqs**	**MeanSqs**	**F.Model**	**R^2**	**adjusted P value**
**Study Group**	1	1.116	1.1158	5.8599	0.02192	0.060
**Sample Day**	1	0.487	0.4865	2.5552	0.00956	0.045
**Age (months)**	1	5.234	5.2339	27.4883	0.10282	0.045
**Sex**	1	1.035	1.0346	5.4336	0.02033	1.000
**Nr. of ABX**	1	1.251	1.2508	6.7361	0.02457	1.000
**Residuals**	225	41.780	0.1857	0.82080		
**Total**	230	50.902	1.00000			

Abbreviations: Df = degrees of freedom, SumsOfSqs = sequential sums of squares, MeanSqs = mean squares, F.Model = F statistics, R^2 = partial R-squared, Nr. of ABX = Number of different antibiotics.

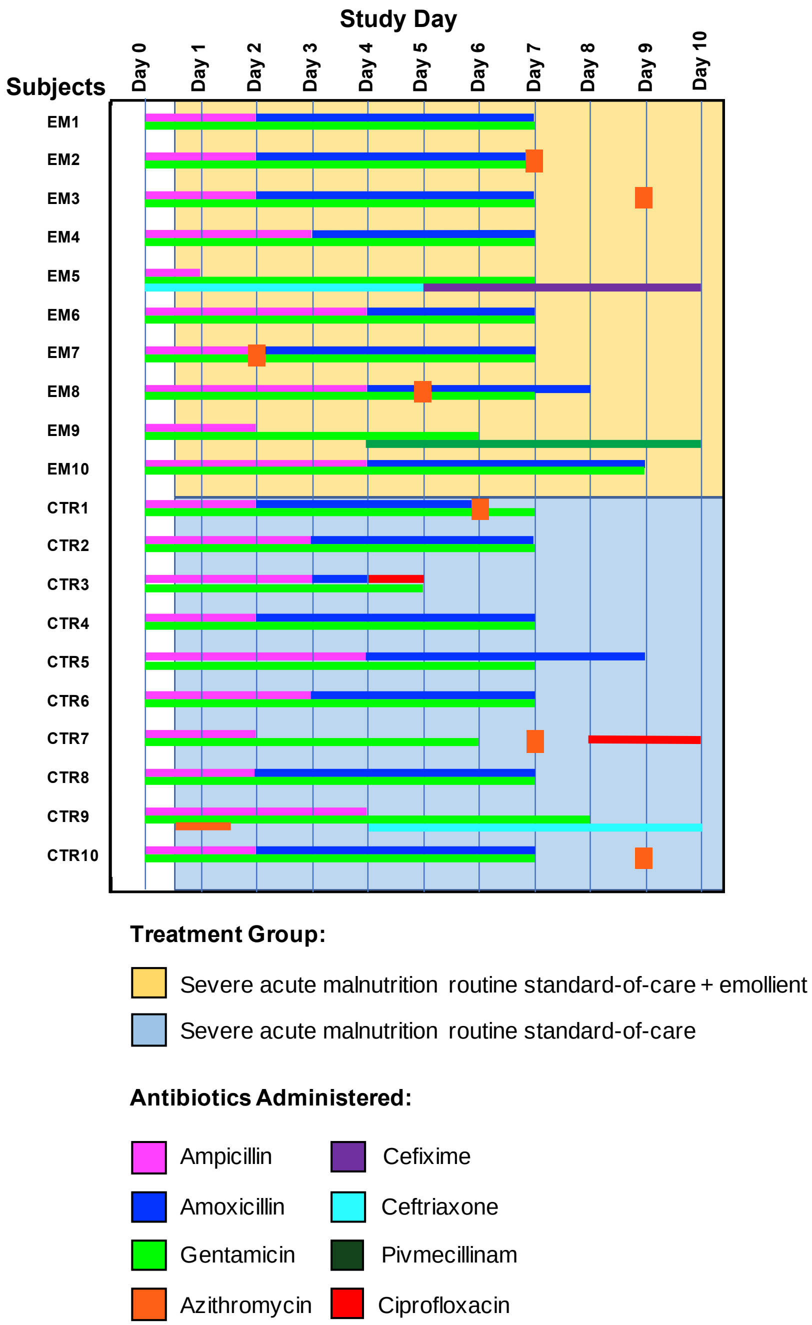

Figure S1. Antibiotic administration during the study time. All study participants received antibiotics after the first sample collection of day 0. Everybody received ampicillin (pink) for the first 3.6 (+/- 1.05) days, followed by amoxicillin (dark blue) for another 5.4 (+/-1.09) days. Additionally, participants received gentamicin (bright green) for the first 7.95 (+/-0.76) days of the study. A few participants received single doses of azithromycin (orange squares) or a short course of cefixime (purple), ceftriaxone (turquoise), pivmecillinam (dark green) or ciprofloxacin (red).

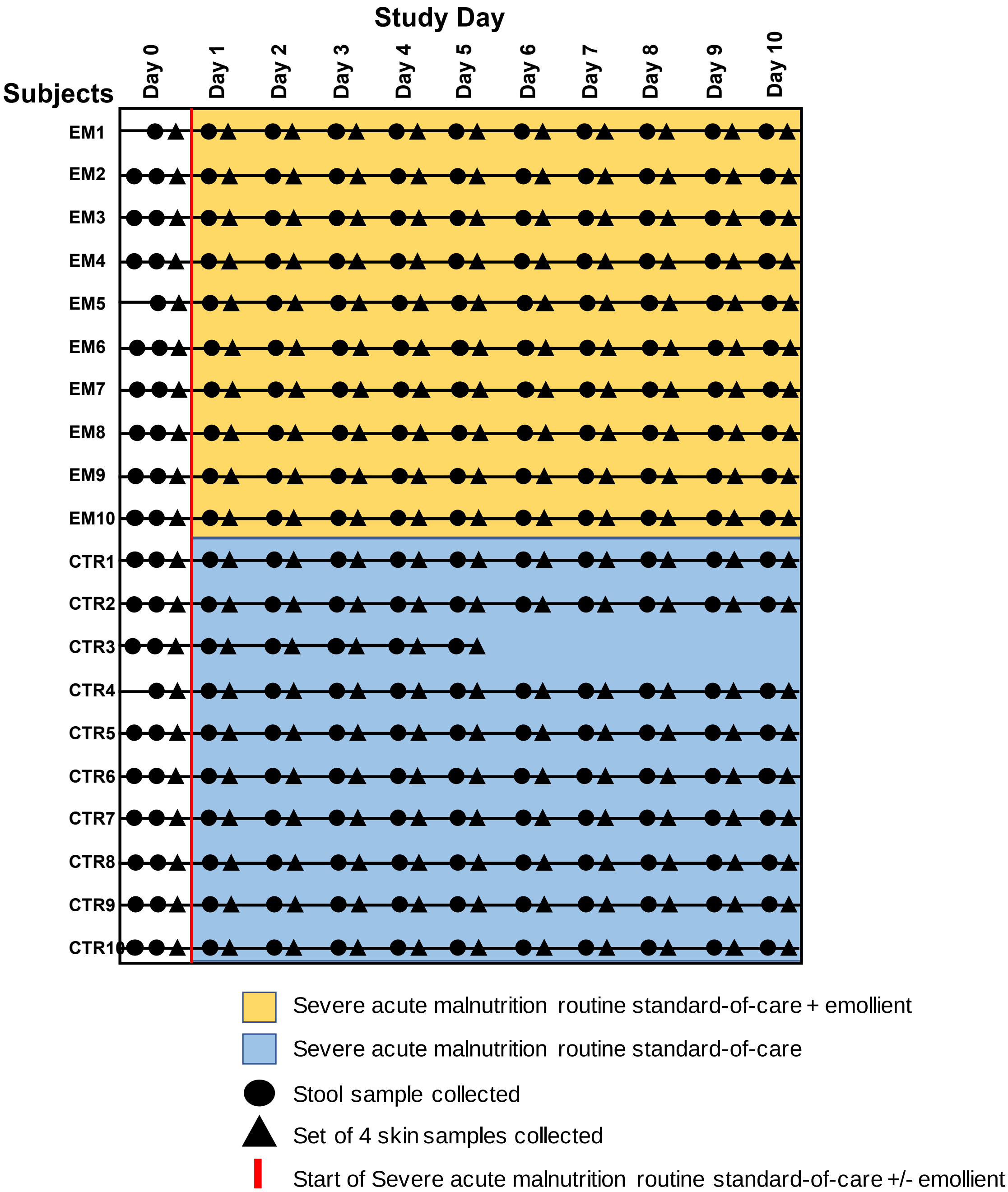

Figure S2. Study design and sample collection scheme. 20 Bangladeshi children of ages 2-18 months with severe acute malnutrition (SAM) were enrolled into the microbiome sub-study upon admission to Dhaka Hospital. One or two stool samples (black circle) and skin swab samples from each of 4 different body sites (black triangle: forehead, volar forearm, elbow crease, shin) were collected at day 0 before the start of any treatment. Of note, participant EM6 had no baseline forehead sample. Participants were then randomized into two treatment groups for days 1-10: i) routine standard-of-care for SAM (blue background); or ii) routine standard-of care for SAM + topical application of high linoleic acid (>60%) SSO whole-body massage, three times daily with 3g of oil per kg of body weight (yellow background). On each of days 1-10 one stool sample and 4 skin swab samples (one from each of the 4 skin sites) were collected from each participant. Participant CTR3 left the hospital after day 5 against medical advice.

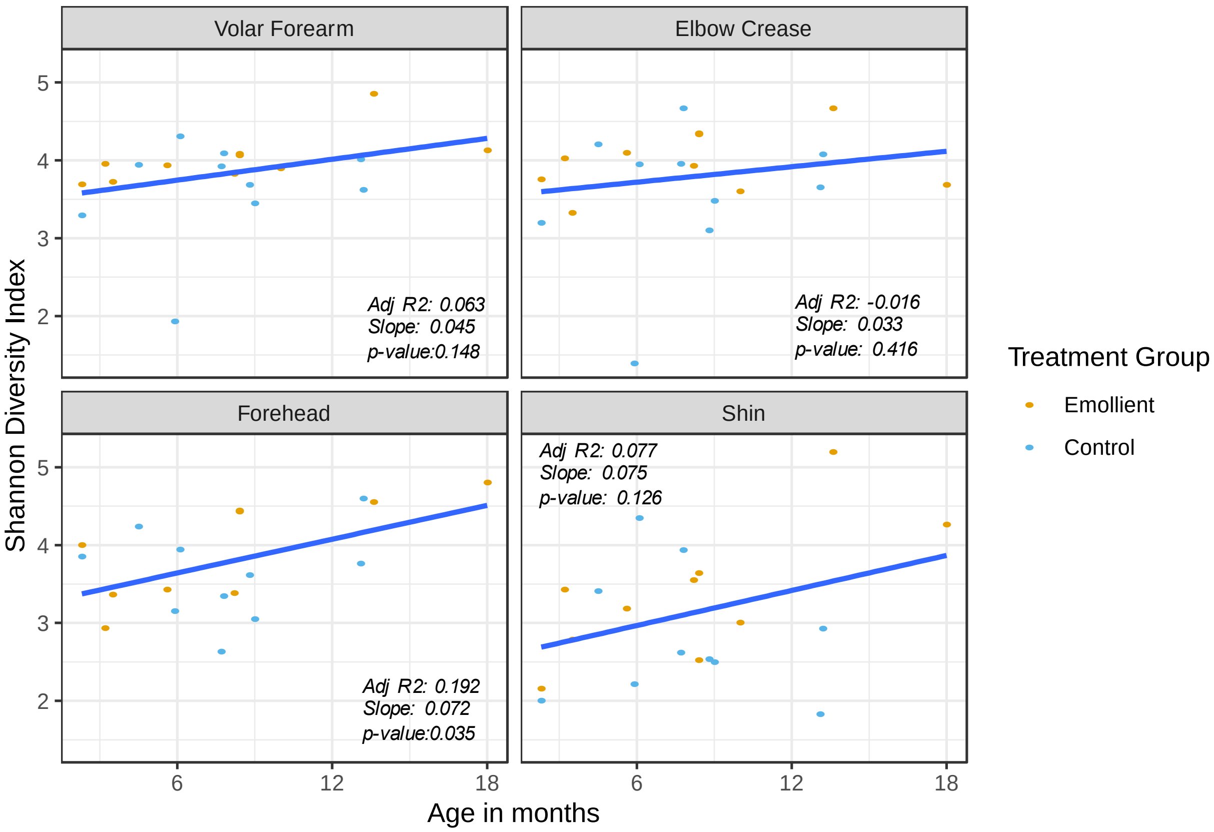

Figure S3. Correlation of Shannon diversity index of the skin microbiota at four body sites with participant age in months. Skin swabs were collected from the forehead (sebaceous habitat), shin (dry habitat), volar forearm (dry habitat) and elbow crease (moist habitat) from 20 Bangladeshi children with SAM of ages 2-18 months, upon admission to Dhaka Hospital. Bacterial DNA was extracted and the 16S rRNA gene was amplified and sequenced. DADA2 was used to identify amplicon sequence variants (ASVs). Mean Shannon diversity index was calculated for each skin site and each subject at the time of enrollment. Linear regression was applied against age in months using the lm function in the stats package in R. Grey shaded area represents the 95% CI. Blue dots = control group, yellow dots = emollient group.

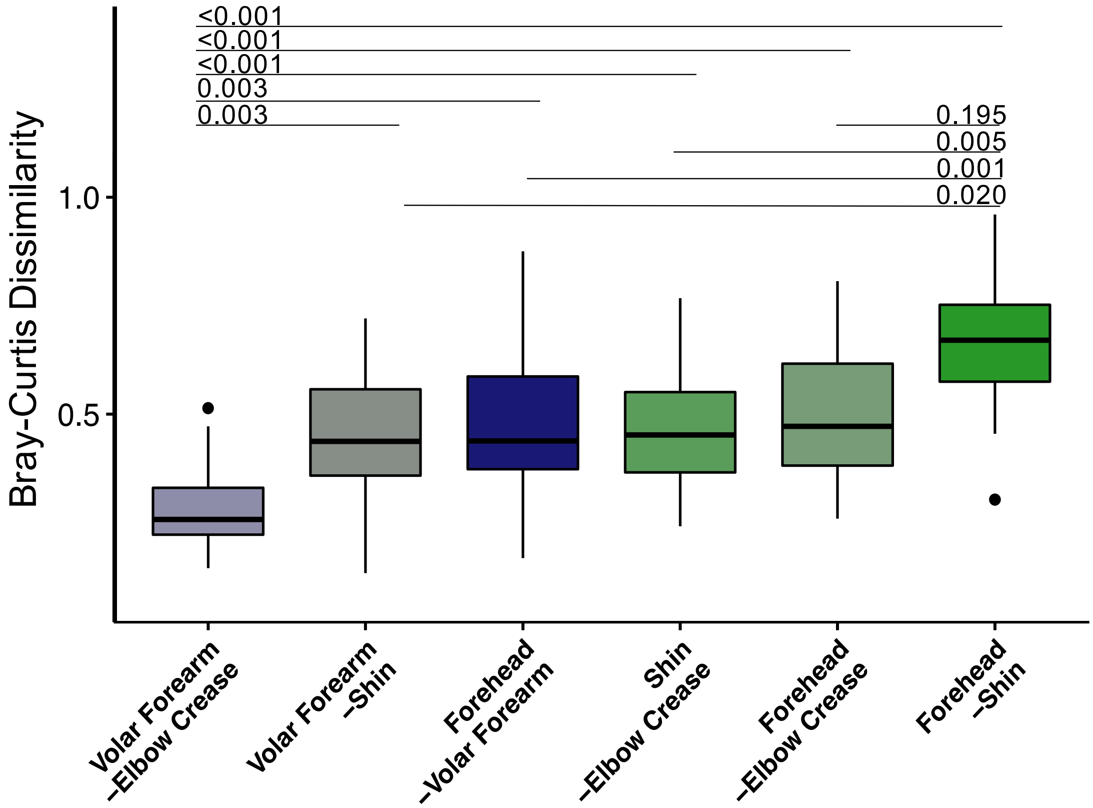

Figure S4. Bray-Curtis dissimilarity between pairs of skin sites within the same child. The Wilcoxon test was performed and the p.adjust function in the stats package (version 3.6.1) was used to adjust for multiple comparisons using the Bonferroni method.

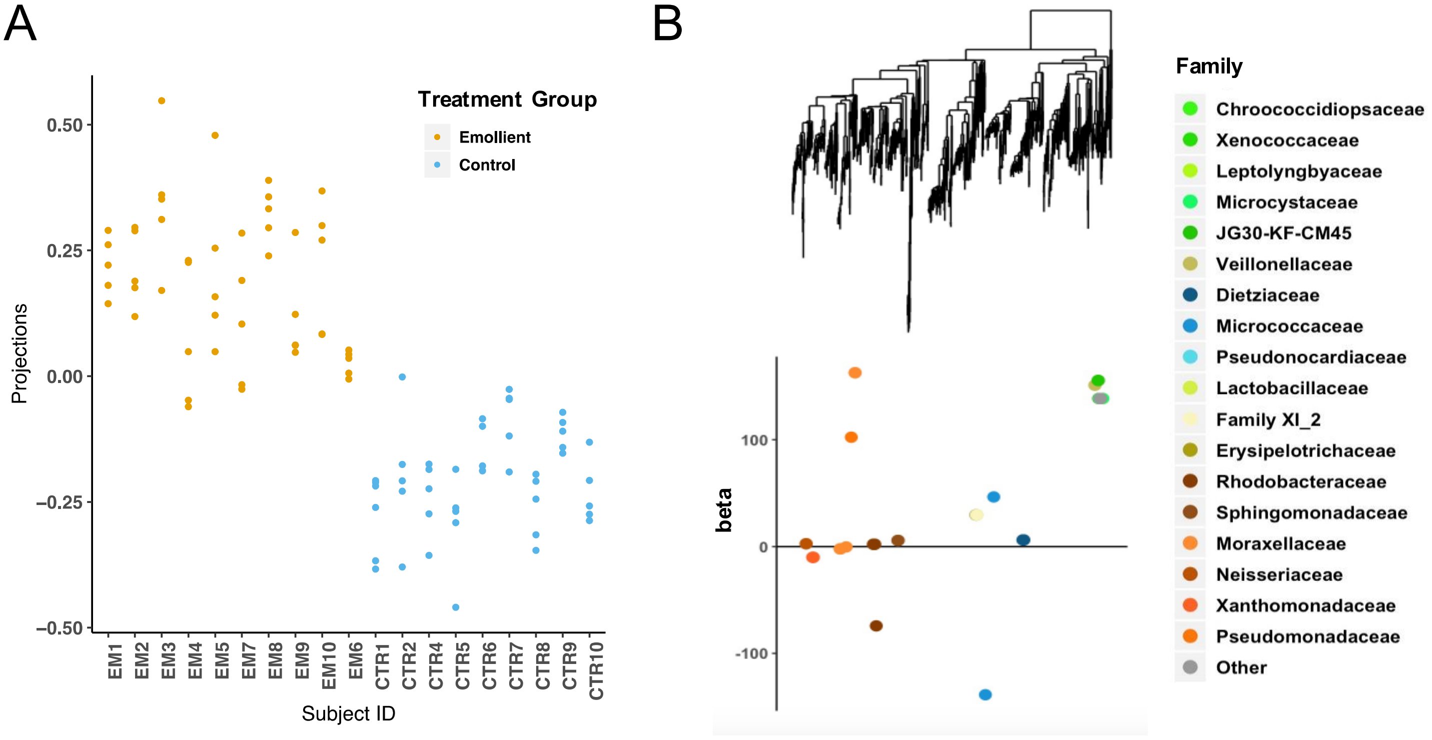

C

Emollient Therapy			Control		
Highest common phylogeny	Nr. Of Member taxa	LDA Coefficient	Highest common phylogeny	Nr. Of Member taxa	LDA Coefficient
Moraxellaceae (f)	2	163	Micrococcus (g)	1	-139
Thermomicrobiales (o)	3	156	Rhodobacteraceae (f)	1	-74
Veillonella (g)	2	151	Stenotrophomonas (g)	4	-10
Bacteria (d)	18	139	Acinetobacter (g)	3	-2
Pseudomonadaceae (f)	1	102			
Micrococcaceae (f)	1	47			
Firmicutes (p)	5	30			
Dietzia (g)	5	6			
Neisseria (g)	2	3			
Rhodobacteraceae (f)	7	2			

Figure S5. Tree-based sparse discriminant analysis of skin microbiota at the volar forearm. Sparse discriminant analysis from the treeDa package (version 0.0.4) was used to identify taxa present in at least 25% of participants on days 6-10 (1305 taxa) that discriminated between treatment groups. After cross-validation 16 predictors were chosen, which corresponded to 59 leaves on the tree. A) Scores of multiple time point samples from days 6- 10 per participant and treatment group on the discriminating axis. B) Taxa loadings on the discriminating axis, colored by Family and plotted along the phylogenetic tree. C) Table with sparse discriminant analysis results per study group, ordered by highest discriminant coefficient (d = domain, p = phylum, o = order, c = class, f = family, g = genus).

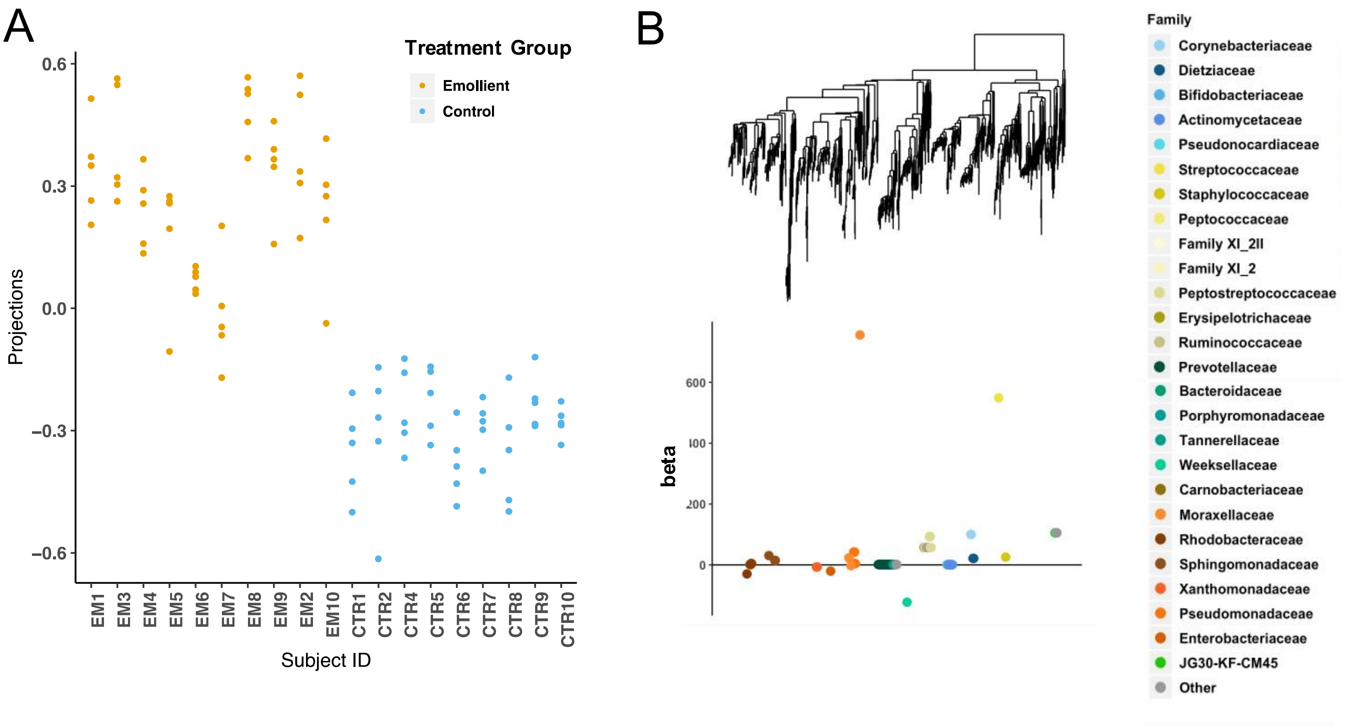

C

Emollient Therapy			Control		
**Highest common phylogeny**	**Nr. Of Member taxa**	**LDA Coefficient**	**Highest common phylogeny**	**Nr. Of Member taxa**	**LDA Coefficient**
**Perlucidibaca (g)**	1	756	**Cruoricaptor (g)**	1	-123
**Lactococcus (g)**	1	549	**Rhodobacteraceae (f)**	1	-29.9
**Bacteria (d)**	8	105	**Enterobacteriaceae (f)**	1	-20.7
**Corynebacterium (g)**	2	99.9	**Stenotrophomonas (g)**	4	-7.02
**Clostridiales (o)**	4	92.9	**Enhydrobacter (g)**	1	-2.6
**Bacteria (d)**	25	57			
**Pseudomonas (g)**	4	42			
**Sphingomonadaceae (f)**	1	30.5			
**Staphylococcus (g)**	1	25.4			
**Acinetobacter (g)**	2	22.9			
**Dietzia (g)**	4	21.3			
**Sphingomonadaceae (f)**	3	14.5			
**Proteobacteria (c)**	11	4.19			
**Bacteroidales (o)**	73	1.01			
**Rhodobacteraceae (f)**	4	1.01			
**Actinobacteria (c)**	22	0.61			

Figure S6. Tree-based sparse discriminant analysis of skin microbiota at the elbow crease. Sparse discriminant analysis from the treeDa package (version 0.0.4) was used to identify taxa present in at least 25% of participants on days 6-10 (1307 taxa) that discriminated between treatment groups. After cross-validation 22 predictors were chosen, which corresponded to 174 leaves on the tree. A) Scores of multiple time point samples from days 6-10 per participant and treatment group on the discriminating axis. B) Taxa loadings on the discriminating axis, colored by Family and plotted along the phylogenetic tree. C) Table with sparse discriminant analysis results per study group, ordered by highest discriminant coefficient (d = domain, p = phylum, o = order, c = class, f = family, g = genus).

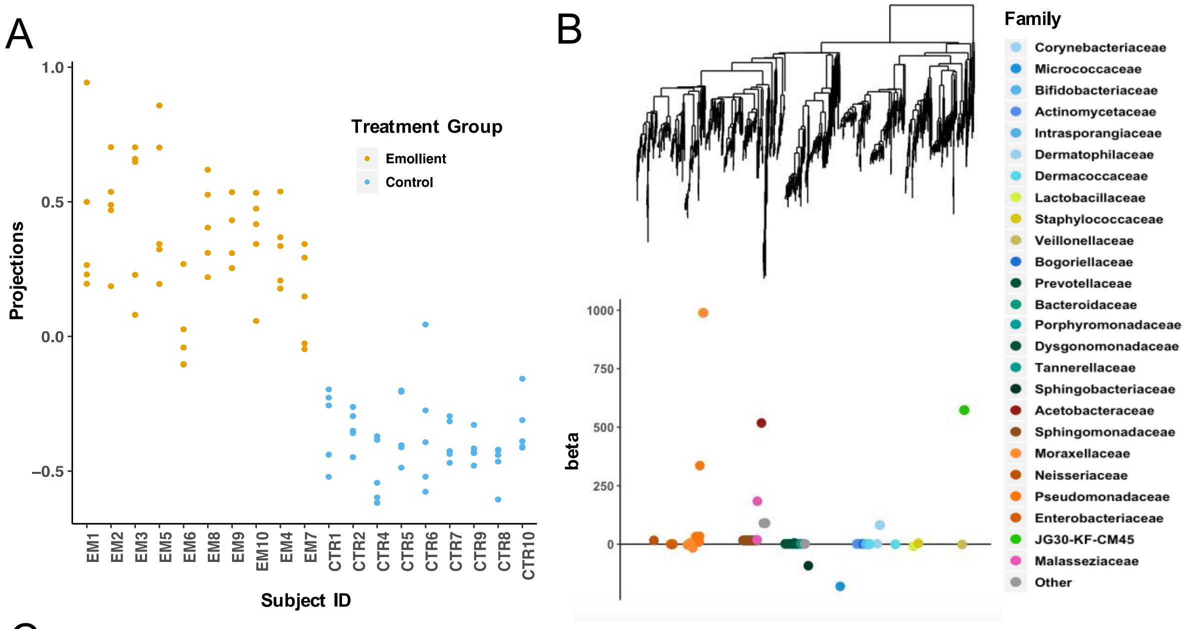

C

Emollient Therapy			Control		
Highest common phylogeny	Nr. Of Member taxa	LDA Coefficient	Highest common phylogeny	Nr. Of Member taxa	LDA Coefficient
Moraxellaceae (f)	3	989	Micrococcus (g)	1	-180
Thermomicrobiales (o)	3	573	Nubsella (g)	1	-92
Endobacter (g)	1	517	Acinetobacter (g)	1	-16.4
Pseudomonadaceae (f)	1	335	Lactobacillus (g)	1	-9.33
Malassezia (g)	1	184	Acinetobacter (g)	3	-2.98
NA	8	90	Veillonella (g)	1	-1.54
Corynebacteriaceae (f)	3	82	Dermacoccaceae (g)	2	-0.55
Pseudomonadaceae (f)	1	33.5	Enterobacteriaceae (f)	8	-0.05
Pseudomonas (g)	4	31.9			
Malassezia (g)	1	19			
Neisseria (g)	1	16.2			
Sphingomonadaceae (f)	44	16			
Pseudomonadaceae (f)	8	9.89			
Prevotella 9 (g)	1	5.24			
Acinetobacter (g)	1	4.96			
Staphylococcus (g)	1	3.53			
Bacteroidales (g)	64	1.25			
Corynebacteriaceae (f)	1	1.22			
Actinobacteria (c)	21	0.91			
Micrococcales (o)	14	0.15			
Acinetobacter (g)	1	0.09			

Figure S7. Tree-based sparse discriminant analysis of skin microbiota at the shin. Sparse discriminant analysis from the treeDa package (version 0.0.4) was used to identify taxa present in at least 25% of participants on days 6-10 (1080 taxa) that discriminated between treatment groups. After cross-validation 29 predictors were chosen, which corresponded to 201 leaves on the tree. A) Scores of multiple time point samples from days 6-10 per participant and treatment group on the discriminating axis. B) Taxa loadings on the discriminating axis, colored by Family and plotted along the phylogenetic tree. C) Table with sparse discriminant analysis results per study group, ordered by highest discriminant coefficient (d = domain, p = phylum, o = order, c = class, f = family, g = genus).

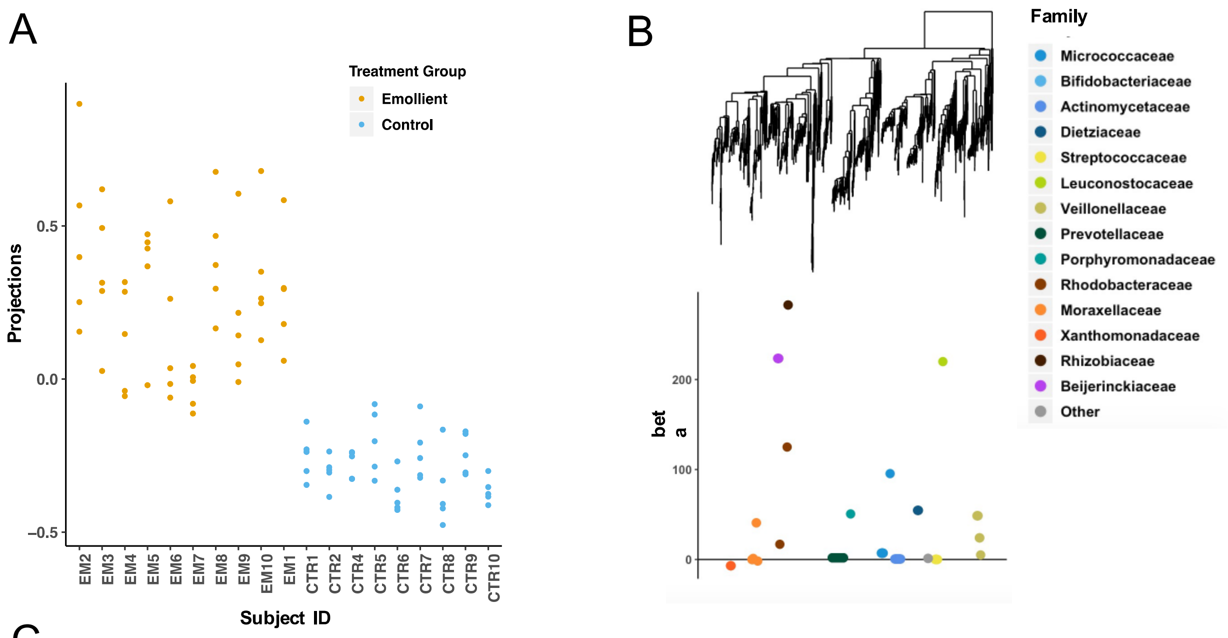

C

Emollient Therapy			Control		
**Highest common phylogeny**	**Nr. Of Member ASVs**	**Coefficient**	**Highest common phylogeny**	**Nr. Of Member ASVs**	**Coefficient**
**Rhizobiaceae (f)**	2	283	**Stenotrophomonas (g)**	4	-6.89
**Methylobacterium (g)**	6	223	**Enhydrobacter (g)**	1	-1.71
**Weissella (g)**	1	220	**Acinetobacter (g)**	4	-0.04
**Rhodobacteraceae (f)**	1	125			
**Microccocaceae (f)**	1	95.4			
**Dietzia (g)**	5	54.6			
**Porphyromonas (g)**	1	50.6			
**Veillonellaceae (f)**	6	48.6			
**Acinetobacter (g)**	2	40.8			
**Veillonella (g)**	3	24			
**Rhodobacteraceae (f)**	1	16.9			
**Microccocaceae (f)**	10	7.1			
**Veillonellaceae (f)**	1	5.09			
**Prevotellaceae (f)**	54	1.89			
**Actinobacteria (c)**	28	0.57			
**Acinetobacter (g)**	1	1.23			
**Actinobacteria (c)**	3	1.23			
**Streptococcus (g)**	9	0.09			
**Streptococcus (g)**	3	0.02			

Figure S8. Tree-based sparse discriminant analysis of skin microbiota at the forehead. Sparse discriminant analysis from the treeDa package (version 0.0.4) was used to identify differential taxa present in at least 25% of participants on days 6-10 (1310 taxa) that discriminated between treatment groups. After cross-validation 22 predictors were chosen, which corresponded to 147 leaves on the tree. A) Scores of multiple time point samples from days 6-10 per participant and treatment group on the discriminating axis. B) Taxa loadings on the discriminating axis, colored by Family and plotted along the phylogenetic tree. C) Table with sparse discriminant analysis results per study group, ordered by highest discriminant coefficient (d = domain, p = phylum, o = order, c = class, f = family, g = genus).

A

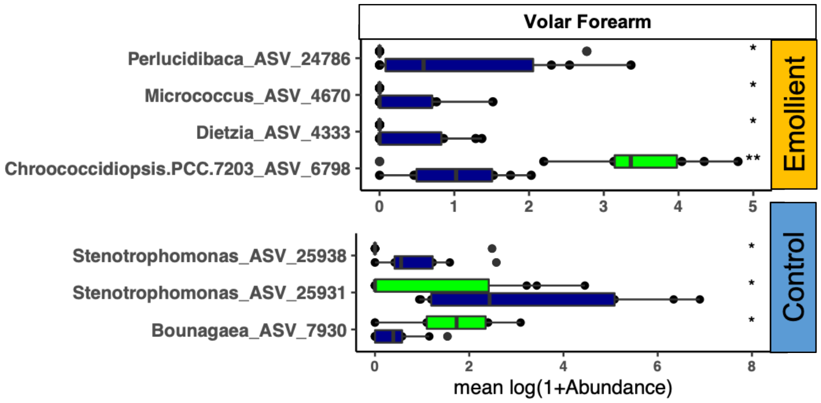
Volar Forearm
Perlucidibaca_ASV_24786
Micrococcus_ASV_4670
Dietzia_ASV_4333
Chroococcidiopsis.PCC.7203_ASV_6798
Emollient
Stenotrophomonas_ASV_25938
Stenotrophomonas_ASV_25931
Bounagaea_ASV_7930
Control
mean log(1+Abundance)

B

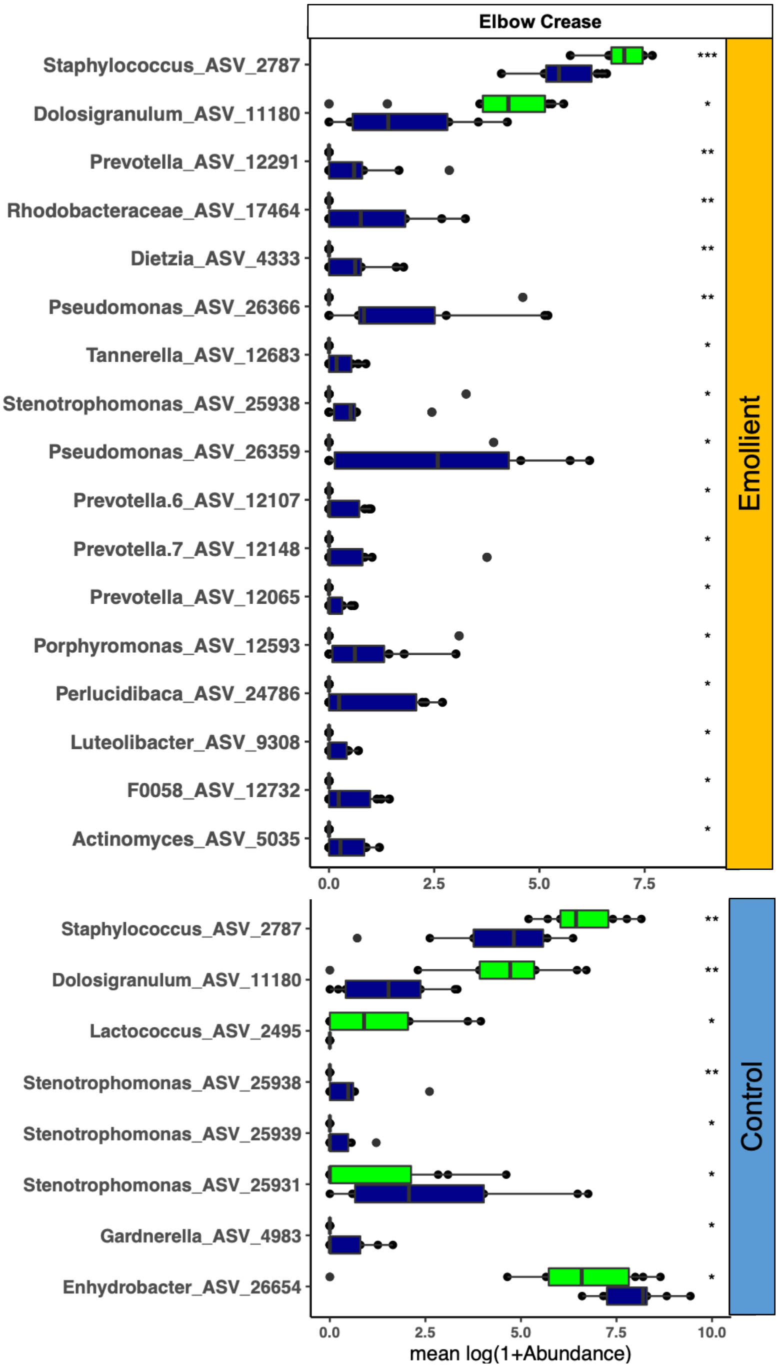
Elbow Crease
Staphylococcus_ASV_2787
Dolosigranulum_ASV_11180
Prevotella_ASV_12291
Rhodobacteraceae_ASV_17464
Dietzia_ASV_4333
Pseudomonas_ASV_26366
Tannerella_ASV_12683
Stenotrophomonas_ASV_25938
Pseudomonas_ASV_26359
Prevotella.6_ASV_12107
Prevotella.7_ASV_12148
Prevotella_ASV_12065
Porphyromonas_ASV_12593
Perlucidibaca_ASV_24786
Luteolibacter_ASV_9308
F0058_ASV_12732
Actinomyces_ASV_5035
Emollient
Staphylococcus_ASV_2787
Dolosigranulum_ASV_11180
Lactococcus_ASV_2495
Stenotrophomonas_ASV_25938
Stenotrophomonas_ASV_25939
Stenotrophomonas_ASV_25931
Gardnerella_ASV_4983
Enhydrobacter_ASV_26654
Control
mean log(1+Abundance)

C

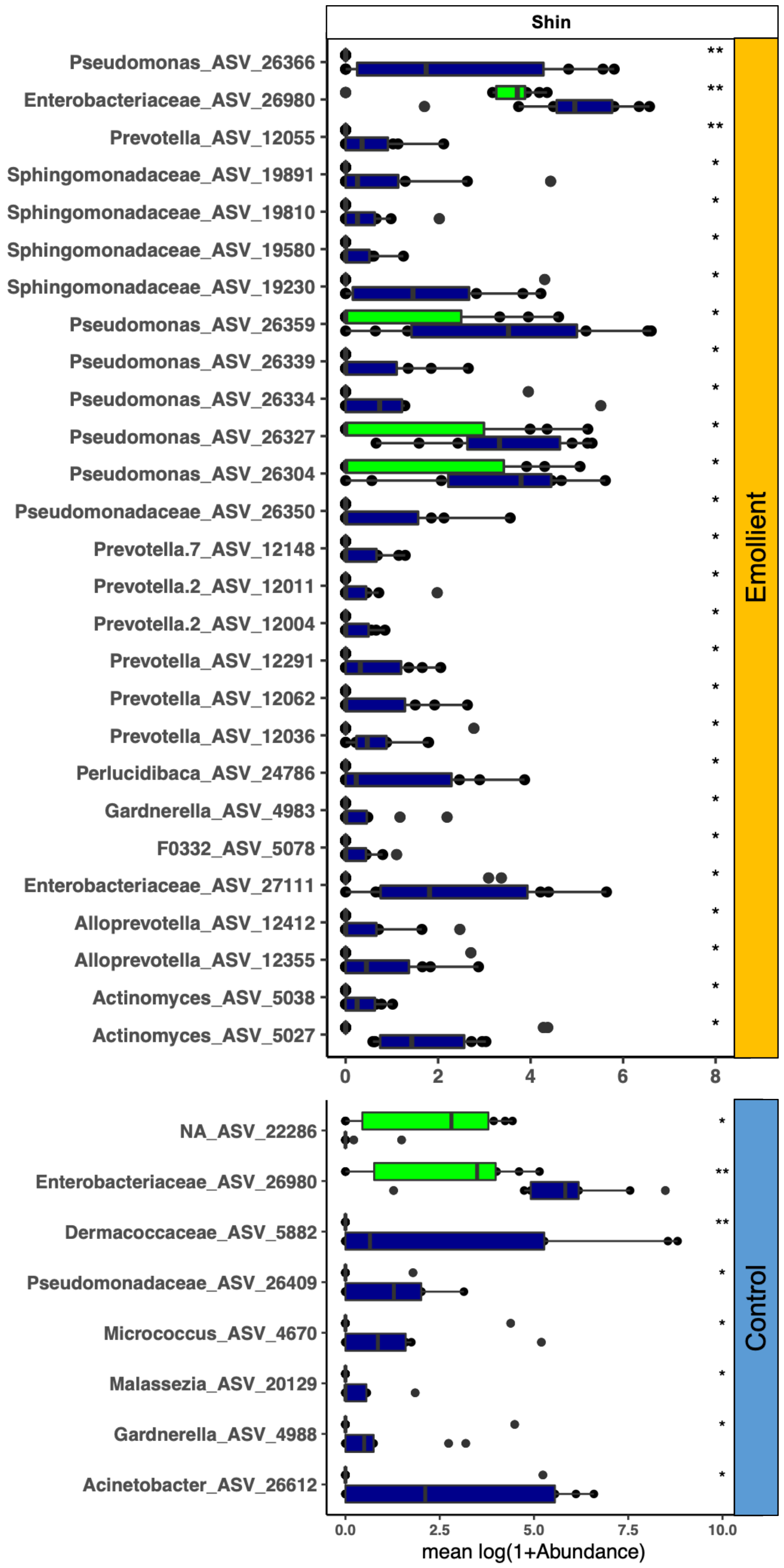

Shin
Pseudomonas_ASV_26366
Enterobacteriaceae_ASV_26980
Prevotella_ASV_12055
Sphingomonadaceae_ASV_19891
Sphingomonadaceae_ASV_19810
Sphingomonadaceae_ASV_19580
Sphingomonadaceae_ASV_19230
Pseudomonas_ASV_26359
Pseudomonas_ASV_26339
Pseudomonas_ASV_26334
Pseudomonas_ASV_26327
Pseudomonas_ASV_26304
Pseudomonadaceae_ASV_26350
Prevotella.7_ASV_12148
Prevotella.2_ASV_12011
Prevotella.2_ASV_12004
Prevotella_ASV_12291
Prevotella_ASV_12062
Prevotella_ASV_12036
Perlucidibaca_ASV_24786
Gardnerella_ASV_4983
F0332_ASV_5078
Enterobacteriaceae_ASV_27111
Alloprevotella_ASV_12412
Alloprevotella_ASV_12355
Actinomyces_ASV_5038
Actinomyces_ASV_5027
Emollient
0 2 4 6 8
NA_ASV_22286
Enterobacteriaceae_ASV_26980
Dermacoccaceae_ASV_5882
Pseudomonadaceae_ASV_26409
Micrococcus_ASV_4670
Malassezia_ASV_20129
Gardnerella_ASV_4988
Acinetobacter_ASV_26612
Control
0.0 2.5 5.0 7.5 10.0
mean log(1+Abundance)

D

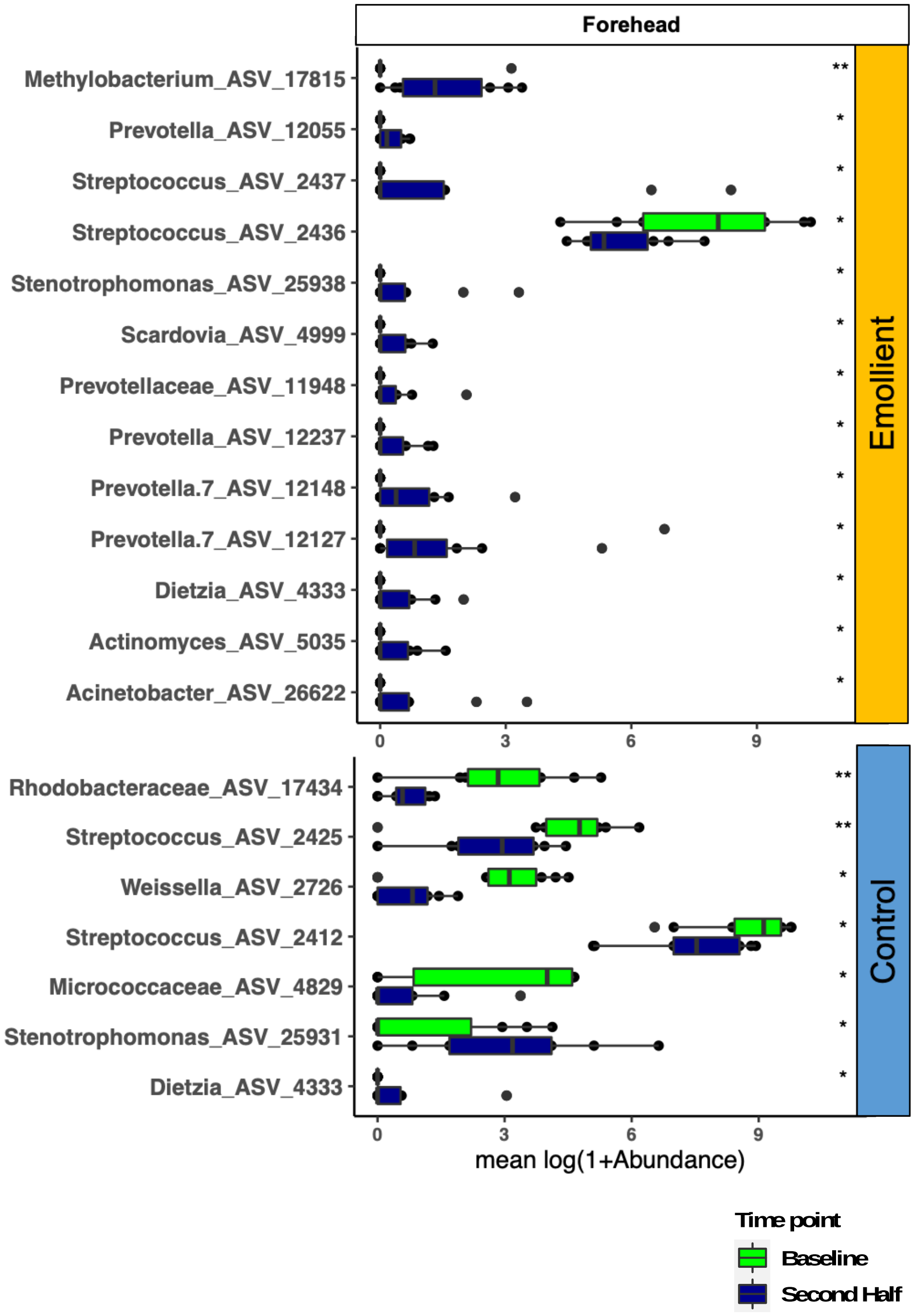

Figure S9. ASVs with significant differences in abundance between baseline and the second half of the study within each treatment group. The mean relative abundance of ASVs identified by LDA as discriminating between the treatment groups, at baseline (green bars) and at days 6-10 (blue bars) was calculated per subject, skin site (A, volar forearm; B, elbow crease; C, shin; D, forehead) and study group. The means were compared using the Wilcoxon test. (P < 0.01**, P < 0.05*). *P* values shown here were not corrected for multiple comparisons. ASVs are presented by skin site and study group.

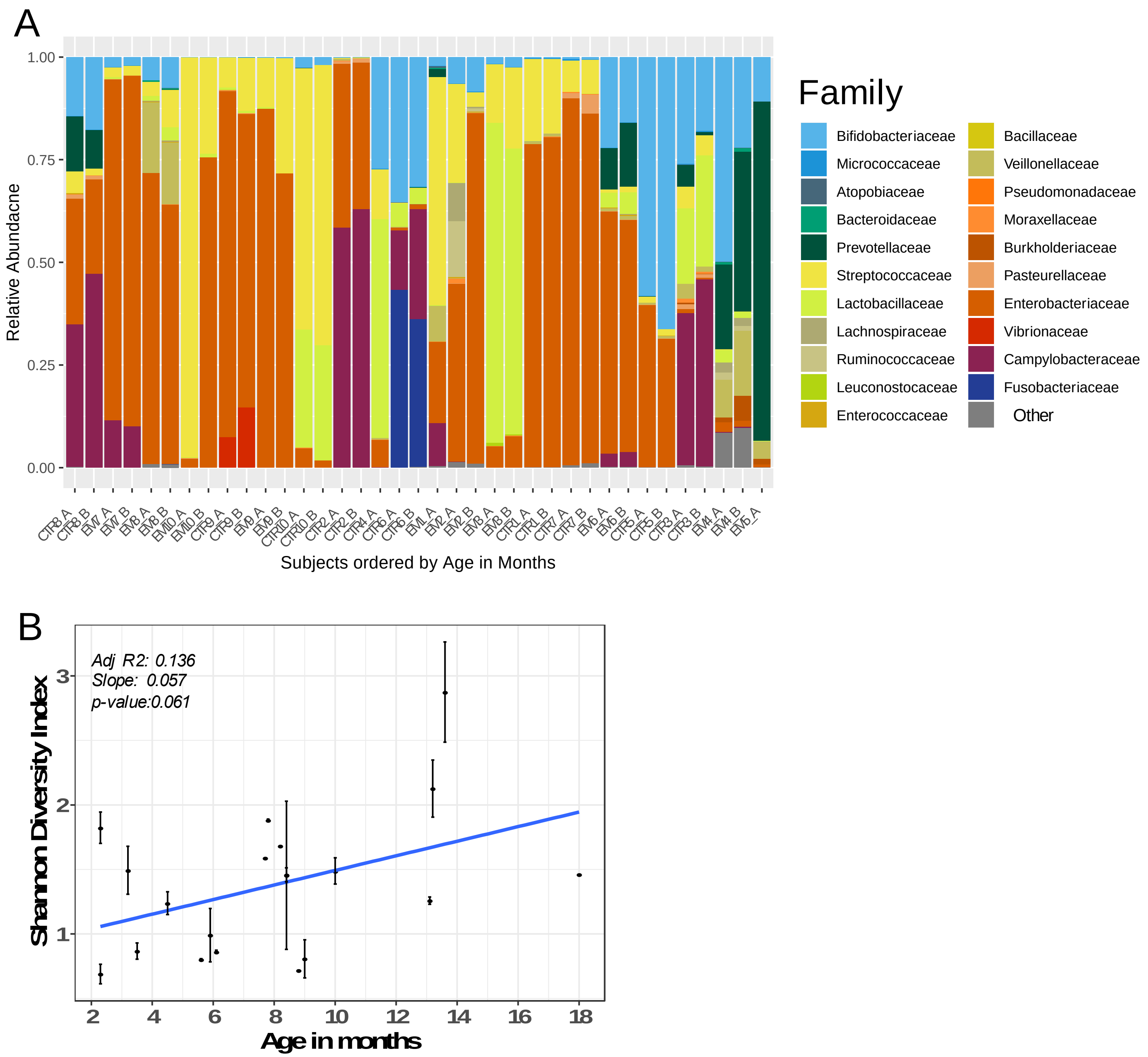

Figure S10. Baseline structure and diversity of the gut microbiota in Bangladeshi children with SAM. Bacterial DNA was extracted from one or two baseline fecal samples of 20 Bangladeshi children with SAM at the time of enrollment. The V4 region of the 16S rRNA gene was amplified, sequenced and sequence variants were inferred using DADA2. Sample counts were transformed into relative abundances and agglomerated at the taxonomic level of Family. A) Structure of the gut microbiota by child, sorted by age in months. Color coded by phylum: shades of blue = Actinobacteria, shades of green = Bacteroidetes, shades of yellow = Firmicutes, shades of red = Proteobacteria, purple: Epsilonbacteraeota, dark blue: Fusobacteria, grey: ASVs with low abundance or without taxonomic assignment. B) Mean Shannon diversity index was calculated across the two baseline samples per subject and displayed with standard deviation. Linear regression was performed using the lm function in the stats package in R on Shannon diversity index versus subject age in months. Grey shaded area represents the 95% confidence interval.
